# Expansion Microscopy Reveals the Spatial Association of Therapeutic Antibody–Receptor Complexes With Cholesterol‐Rich Membrane Domains

**DOI:** 10.1002/anie.1206161

**Published:** 2026-08-24

**Authors:** Gang Wen, Nicole Seifert, Patrick Eiring, Johanna Meyer, Hannes Neuweiler, Julia Weingart, Ignacio Vega‐Vásquez, Sören Doose, Philip Kollmannsberger, Markus Sauer

**Affiliations:** ^1^ State Key Laboratory of Radiation Medicine and Protection School of Radiation Medicine and Protection Collaborative Innovation Center of Radiological Medicine of Jiangsu Higher Education Institutions Soochow University Suzhou P. R. China; ^2^ Department of Biotechnology and Biophysics Biocenter University of Würzburg Würzburg Germany; ^3^ Rudolf Virchow Center Research Center for Integrative and Translational Bioimaging University of Würzburg Würzburg Germany

**Keywords:** CD20, confocal, cytotoxicity, cell biology, lipid raft, monoclonal antibody, signal transduction

## Abstract

Understanding the complex interplay between therapeutic monoclonal antibodies (mAbs) and target receptors within the plasma membrane is essential for improving immunotherapy efficacy. However, direct visualization of lipid nanodomains remains challenging due to their nanoscale dimensions and dynamic behavior. Here, we combine expansion microscopy (ExM) with a fluorescent perfringolysin O domain‐4 probe to map cholesterol‐rich nanodomains in the membrane of whole intact cells with a spatial resolution approaching ∼40 nm on a confocal setup. We demonstrate that cholesterol‐rich domains predominantly localize to actin‐supported membrane protrusions in COS‐7 cells. We directly visualize how the binding of therapeutic mAbs rituximab (RTX) and daratumumab (DARA) redistributes CD20 and CD38 on lymphoma and multiple myeloma cells, respectively, into cholesterol‐rich nanodomains. Accumulation of receptor/mAb complexes within lipid nanodomains creates Fc fragment‐dense regions that enhance signal transduction, apoptotic signaling, and complement‐dependent cytotoxicity. Furthermore, RTX binding induces pronounced B‐cell polarization and accumulation of CD20/RTX complexes in cholesterol‐rich membrane nanodomains, whereas CD38/DARA complexes exhibit a more homogeneous membrane distribution indicating that mAb binding‐induced receptor reorganization occurs in a cell‐type‐ and receptor‐dependent manner at the nanoscale. Our results show how ExM can be used advantageously to improve our understanding of the complex interplay of mAbs and lipid nanodomains.

## Graphical abstract

We report a super‐resolution strategy that maps cholesterol‐rich membrane domains in whole, intact cells with an effective spatial resolution of ∼40 nm by combining expansion microscopy with a perfringolysin O domain 4 probe. This approach enables direct visualization of the spatial association between CD20 and CD38 complexes and cholesterol‐rich membrane domains following treatment with the therapeutic antibodies rituximab and daratumumab.

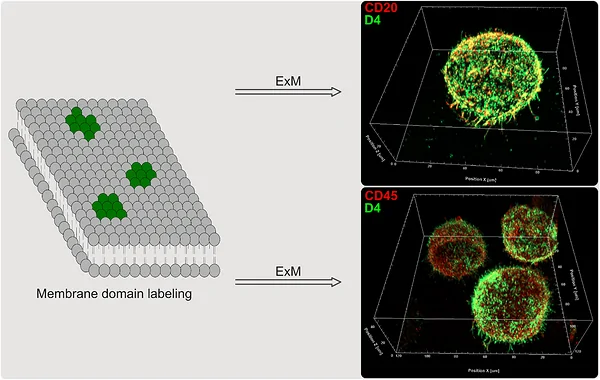

## 1 Introduction

The cell membrane, a barrier that separates its internal environment from the outside, is more than a passive barrier, but a dynamic mosaic of lipids and proteins that coordinate cell signaling [1, 2]. While early models like the Singer–Nicolson fluid mosaic model described membranes as homogenous lipid bilayer with proteins freely diffusing [3], subsequent discoveries revealed that lipids are not uniformly distributed in the membrane. Instead, membranes contain lipid nanodomains or lipid rafts, enriched in cholesterol and sphingolipids, such as sphingomyelin and glycosphingolipids [1, 2, 4, 5]. These lipid nanodomains (typically on the order of 10–200 nm) are dynamic assemblies of specific lipids and proteins that float within the more fluid bulk membrane and can coalesce into larger platforms [1, 4, 5, 6, 7]. By concentrating certain receptors and other molecules, lipid rafts serve as specialized signaling platforms that play a critical role in the regulation of various cellular processes, including signal transduction [8, 9], membrane trafficking/organization [2, 5, 10], and protein sorting [11, 12].

Because of their dynamic nature, the existence of lipid nanodomains in cell membranes has been controversially discussed over the past decades [6, 13]. Recently, several emerging biochemical and biophysical techniques have provided support for the presence of these domains in cells [14], including fluorescence resonance energy transfer (FRET) measuring the proximity of lipid raft components [15, 16], and Stimulated Emission Depletion (STED)‐FCS disclosing that sphingolipids and GPI‐anchored protein analogs exhibit transient trapping for several milliseconds in cholesterol‐dependent sites that are smaller than 80 nm [17, 18]. Direct visualization of lipid nanodomains in cell membranes has been demonstrated by freeze‐fracture electron microscopy and immunogold labeling revealing heterogenous membrane structures consistent with lipid nanodomains or rafts [19]. Using Photo‐Activated Localization Microscopy (PALM) of raft and non‐raft targeting proteins in combination with pair‐correlation analysis identified clusters in cell membranes on a scale of 50–100 nm [20, 21], and live‐cell STED microscopy with a functionalized cholesterol probe demonstrated the existence of cholesterol‐rich membrane nanodomains of <50 nm [22]. However, lipid rafts continue to elude direct microscopic detection and remain thus subject of debate, particularly as different methodologies can yield seemingly contradictory results [23]. For example, labeling of lipids can prevent them from entering lipid rafts, or induce artificial clustering [19, 24].

Despite these inconsistencies, there is proven evidence that lipid rafts play important roles in various diseases including neurodegenerative diseases, cardiovascular diseases, and cancer [25, 26]. By clustering receptors and associated signaling proteins, rafts can amplify or modulate signal transduction, thereby influencing how cancer cells respond to external stimuli and therapeutic agents [27]. Since many cell surface receptors aggregate within lipid rafts, their localization can affect how therapeutic antibodies bind to tumor‐associated receptors and initiate downstream signaling. Although therapeutic monoclonal antibodies (mAbs) have been an essential pillar of cancer immunotherapy, knowledge about their molecular interaction with receptors, that is, their operating mode remains elusive. Open questions include how binding of mAbs induces tumor cell death by various mechanisms, and how these processes are related to the localization of receptors in lipid rafts [28, 29]. Binding of mAbs to receptors can eliminate tumor cells through multiple mechanisms including antibody‐dependent cellular cytotoxicity (ADCC) or phagocytosis (ADCP), complement‐dependent cytotoxicity (CDC), and direct triggering of cell death [30]. Here, rituximab (RTX) plays an important role as it was first approved in 1994 and still serves as reference mAbs for the treatment of B cell related malignancies [31]. RTX is directed against CD20 and after binding and crosslinking of CD20 reported to induce a redistribution of CD20 into low‐density detergent‐insoluble membrane compartments [32, 33, 34]. However, the direct visualization of CD20 in lipid rafts after mAb treatment has failed yet.

Because the refined understanding of the interaction between lipid nanodomains and mAbs is crucial for the improvement of immunotherapy efficacy, we set out to visualize the localization of lipid nanodomains and tumor‐associated receptors in the absence and presence of mAbs on whole cells at the nanoscale by expansion microscopy (ExM) [35]. ExM has revolutionized fluorescence imaging because it allows multicolor super‐resolution fluorescence imaging of proteins, lipids, and nucleic acids in cells on standard microscopes [36, 37, 38, 39, 40, 41], and should thus enable to study the colocalization of lipid nanodomains and receptors. While some details of the molecular operating principle of RTX and CD20 on B cells have been revealed in recent years [30, 42, 43], the molecular interaction between CD38 and daratumumab (DARA), the first CD38‐targeting mAb approved for the treatment of multiple myeloma (MM) in 2015, has been less intense investigated [29, 44]. Therefore, we used CD38 as a reference system to investigate the mAb‐binding induced colocalization of tumor‐associated receptors with lipid nanodomains.

## 2 Results and Discussion

### 2.1 Visualizing Cholesterol‐Rich Membrane Domains by ExM

To date, either functionalized lipids [22, 23, 39, 45, 46], or lipid‐binding proteins [47, 48, 49, 50], have been used for direct visualization of membrane domains. Because functionalized lipids must be modified with an anchoring group for covalent linkage into hydrogels [39], we used the fluorescently labeled domain 4 (D4) of perfringolysin O, a non‐cytotoxic cholesterol‐binding toxin that can be efficiently linked into hydrogels by its lysine residues (Figure 1) [50, 51, 52]. EGFP‐tagged D4 (EGFP‐D4) was expressed in bacteria and used for cholesterol staining in COS‐7 cells at 4°C to avoid internalization. Reference experiments where cells have been treated with methyl‐ß‐cyclodextrin (MßCD) to deplete cholesterol [5, 53], showed no EGFP‐D4 staining, confirming that EGFP‐D4 binding is cholesterol‐dependent (Figure S1). As it has been reported that cell permeabilization is crucial for isotropic expansion of cells [35, 38], we investigated the binding performance of EGFP‐D4 on Triton X‐100 and saponin treated cells, respectively. The results obtained demonstrate that EGFP‐D4 can be fixed and well retained after application of standard fixation procedures (Figure S2). Compared to other super‐resolution microscopy techniques such as *direct* stochastic optical reconstruction microscopy (*d*STORM) and stimulated emission depletion (STED) microscopy, ExM readily enables multicolor nanoscale imaging of membrane domains on whole cells using confocal microscopes. In addition, ExM uses common organic dyes or fluorescent proteins and requires minimal technical expertise [36]. Therefore, we next tested the performance of EGFP‐D4 for imaging of cholesterol‐rich lipid domains in cells by ExM. Here, we used Acryloyl‐X (AcX) to link lysine residues of proteins into the hydrogel (Figure 1) [38].

**FIGURE 1:**
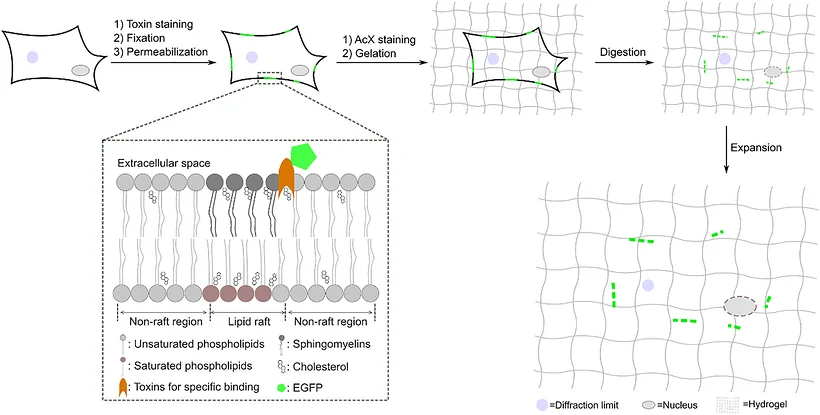
Schematic diagram of the visualization of membrane domains by ExM. Cholesterol‐rich membrane domains were labeled with fluorescently labeled domain 4 (D4), fixed, permeabilized, and crosslinked into the hydrogel with acryloyl‐X (AcX) followed by gelation, digestion, and expansion. The black line indicates cell membranes.

By comparing cholesterol‐rich membrane domains before and after expansion in the same cell, we determined an expansion factor of 4.10 ± 0.10 (mean ± s.d., *n* = 60) (Figure S3). After 4.1‐fold expansion, lipid domains were clearly visible at the nanoscale (Figure 2a–f) and domains that cannot be distinguished by confocal microscopy were easily resolved after expansion (Figure 2g). Three‐dimensional (3D) confocal images revealed that cholesterol‐rich membrane domains labeled with EGFP‐D4 are predominantly located on the cellular surface (Figure 2h and Movie S1). To further increase the image resolution, we used Airyscan microscopy, allowing us to resolve cholesterol‐rich membrane domains with sizes of ∼40 nm, in agreement with previous reports (Figures 2i–k, and Movie S2) [54, 55]. This allowed us to estimate the apparent size of EGFP‐D4‐positive puncta to 64 ± 17 nm (mean ± s.d., *n* = 320) (Figure S4).

**FIGURE 2:**
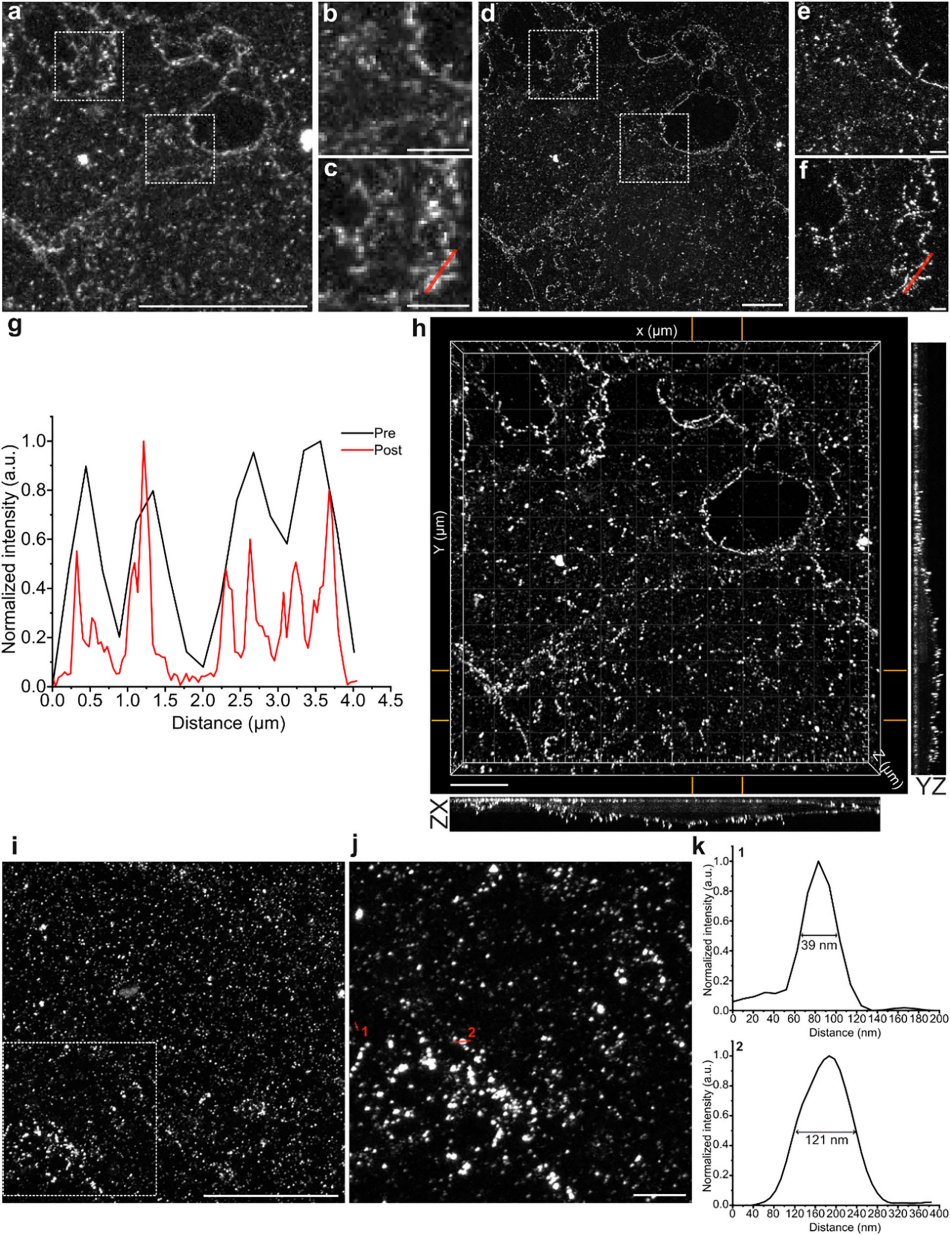
Visualizing cholesterol‐rich membrane domains in COS‐7 cells by 4x ExM. (a to f) Comparison of confocal images of the same cholesterol‐rich membrane domains before (a–c) and after 4x ExM (d–f). (g) Fluorescence intensity profiles along the red lines in (c and f), emphasizing the higher resolution achieved after 4.1‐fold expansion. (h) Corresponding 3D image of cholesterol‐rich membrane domains. (i, j) Airyscan super‐resolution image of cholesterol‐rich membrane domains (i) and zoom‐in view of the highlighted region (j). (k) Normalized fluorescence intensity profiles along the red lines in (j). The distances correspond to the pre‐expansion dimension, adjusted according to the expansion factor. Representative images were selected from n = 3 independent samples. Scale bars, 30 µm (a, d, h, and i) and 5 µm (b, c, e, f, and j).

Even though 3D images of expanded cells suggest that EGFP‐D4 labeled cholesterol‐rich membrane domains are located in the outer cellular membrane (Figure 2h), we performed colocalization experiments with different vesicle markers to exclude that the signals we observed originate from the internalization of EGFP‐D4. However, neither clathrin‐coated pits nor Rab 5A‐enriched early endosomes [56, 57], or LAMP1‐enriched late endosomes/lysosomes [58], colocalized with the EGFP‐D4 signal (Figure S5–S7), confirming that the signals detected after 4.1‐fold expansion represent cholesterol‐rich membrane domains. Comparing the same cellular features before and after expansion demonstrates that ExM allows a more accurate quantification of colocalization coefficients (Figure S6). In addition, we performed four‐color ExM of cholesterol‐rich membrane domains co‐stained with antibodies directed against clathrin‐coated pits, early endosomes, and DAPI to visualize nucleic acids. The resulting Airyscan images visualized endosomes and hollow clathrin‐coated pits but did not exhibit colocalization with EGFP‐D4 at the nanoscale (Figure S8 and Movie S3).

### 2.2 Cholesterol‐Rich Membrane Domains Accumulate on Microvilli‐Like Protrusions

Closer examination of ExM images revealed that cholesterol‐rich membrane domains accumulate on membrane protrusions of COS‐7 cells (Figure 3a–c and Movie S4). Actin‐based protrusions are essential for many fundamental cellular processes, including sensing of the environment, cell adhesion, migration, cancer metastasis, immunological synapse formation, and cell‐cell fusion [59, 60]. Since the observed protrusions exhibit a diameter in the range of 50–500 nm and a length of several micrometers (Figure S9) we identified them as microvilli‐like protrusions [61]. Microvilli are finger‐like membrane protrusions that are found on the surface of a wide variety of cell types and supported by actin filaments (F‐actin) that are organized in parallel bundles of 10–30 filaments [61]. To verify the observation that cholesterol‐rich membrane domains accumulate on microvilli, we labeled F‐actin using a multifunctional phalloidin linker developed for ExM applications [62, 63]. The resulting ExM images consistently demonstrated that cholesterol‐rich membrane domains frequently accumulate on microvilli‐like protrusions (Figures 3d–f, S10 and S11, and Movies S5 and S6) [64, 65, 66].

**FIGURE 3:**
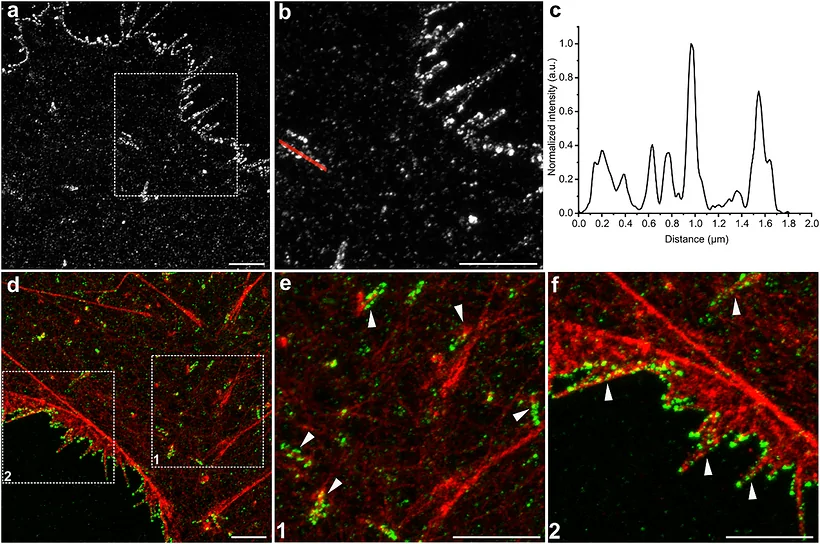
Cholesterol‐rich membrane domains colocalize with actin filaments. COS‐7 cells were stained with EGFP‐D4 at 4°C, fixed, permeabilized, stained with multifunctional phalloidin linkers and expanded using the procedure of 4x ExM. (a, b) Airyscan super‐resolution images of cholesterol‐rich membrane domains in microvilli‐bearing cells imaged with an 63x oil immersion objective (a) and zoom‐in view of the highlighted region (b). (c) Normalized fluorescence intensity profile along the red line in (b). The distance corresponds to the pre‐expansion dimension, adjusted according to the expansion factor. (d) Airyscan super‐resolution image of cholesterol domains (EGFP‐D4, green) and actin (‘Actin ExM’, 561, red) highlighting the accumulation of cholesterol‐rich membrane domains on microvilli of COS‐7 cells. (e, f) Zoom‐in views of the highlighted regions in panel d, showing protruding and sandwiched microvilli between the basal membrane and the coverslip. Representative images were selected from *n* = 3 independent samples. Scale bars, 10 µm.

### 2.3 CD20/RTX and CD38/DARA Complexes Colocalize With Cholesterol‐Rich Membrane Domains

Recently, it has been shown that the binding of the therapeutic monoclonal antibody (mAb) RTX induces polarization of B cells, crosslinking of homodimeric CD20 and strong accumulation of the RTX/CD20 signal on microvilli [43]. As a type I mAb, it is anticipated that RTX binding activates complement‐dependent cytotoxicity (CDC) by crosslinking of CD20 receptors and the resulting oligomerization of the mAb's Fc region to increase the binding avidity of complement component 1q (C1q), which is a hexa‐headed molecule that optimally binds hexameric Fc arrangements [42, 67]. mAb binding‐induced redistribution and accumulation of CD20 in lipid nanodomains as suggested by different studies, might thus promote the formation of Fc‐dense regions improving signal transduction, apoptotic signaling, and recruitment of C1q [32, 33, 34, 68]. However, due to the lack of suitable lipid nanodomain probes and the limited spatial resolution of standard imaging methods, direct visualization of the colocalization of lipid nanodomains and RTX‐induced CD20 clustering on whole cells remained challenging.

To address this problem, we co‐stained living Raji B cells with EGFP‐D4 and RTX labeled with CF568 for 15 min at 37°C before fixation to allow crosslinking and redistribution of CD20/RTX complexes into lipid nanodomains. 3D Airyscan images of expanded B cells clearly demonstrated the colocalization of cholesterol‐rich membrane domains and CD20/RTX clusters on the membrane, particularly, on microvilli protrusions (Figures 4a–e, S12, and S13, and Movie S7). In addition, quantitative analysis of fluorescence intensity distributions revealed a pronounced enrichment of RTX/CD20 complexes and cholesterol‐rich membrane domains in polarized membrane regions as has been reported previously (Figures S14 and S15) [43]. Interestingly, the binding of the EGFP‐D4 probe alone already induced polarization of some B cells, although less frequently than observed in RTX‐treated cells (Figures S13 and S16).

**FIGURE 4:**
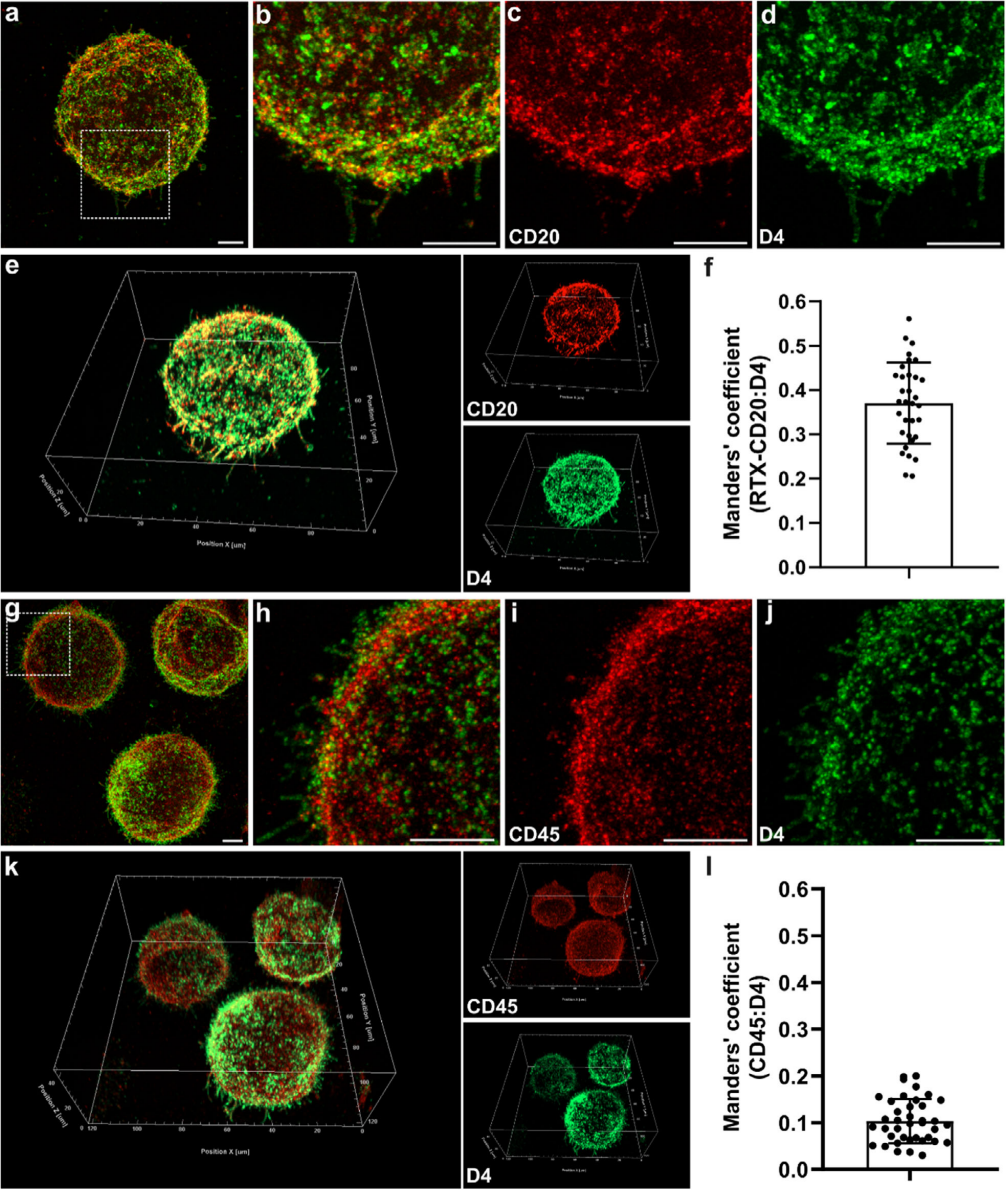
RTX binding to CD20 on B cells induces redistribution of CD20/RTX complexes into cholesterol‐rich membrane domains and accumulation on microvilli‐like protrusions. Raji B cells were labeled with EGFP‐D4 and CF568‐labeled mAbs for 15 min, fixed, permeabilized, and expanded. (a–d) Airyscan super‐resolution images of RTX and cholesterol‐rich membrane domains (a) and a zoom‐in view of the boxed region (b‐d) after expansion. Cholesterol‐rich membrane domains (EGFP‐D4) are shown in green and RTX‐stained CD20 (CF568) in red. (e) Corresponding 3D image of RTX and cholesterol‐rich membrane domains. (f) Analysis of whole cell data yields a colocalization coefficient of RTX and cholesterol‐rich membrane domains on B cells of 0.370 ± 0.092 (mean ± s.d., *n* = 33 cells). “RTX‐CD20:D4” represents the fraction of RXT‐CD20 signal overlapping with EGFP‐D4. (g‐j) Airyscan super‐resolution image of CD45 (red) and cholesterol‐rich membrane domains (green) (g) and a zoom‐in view of the boxed region (h‐j) on B cells. (k) Corresponding 3D image of anti‐CD45 mAb and cholesterol‐rich membrane domains. (l) Analysis of whole cell data yields a colocalization coefficient of anti‐CD45 mAb and cholesterol‐rich membrane domains on B cells of 0.103 ± 0.047 (*n* = 39 cells). “CD45:D4” represents the fraction of CD45 signal overlapping with EGFP‐D4. Representative images were selected from *n* = 4 (a‐e) or *n* = 3 (g–k) independent samples. Scale bars, 10 µm.

Using Mander's analysis for quantification of the colocalization in whole Z‐stacks [46, 69], we obtained an average colocalization coefficient of 0.370 ± 0.092 of CD20/RTX clusters overlapping with cholesterol‐rich membrane domains on B cells (Figure 4f).

As a control reference, we used CD45, a transmembrane protein that is described to be largely excluded from lipid nanodomains and tips of microvilli [43, 68, 70, 71]. Consistent with previous reports, we measured a low Manders’ colocalization coefficient of 0.103 ± 0.047 for CD45 overlapping with cholesterol‐rich membrane domains [70]. In addition, CD45 did not accumulate on microvilli‐like protrusions (Figures 4g–l, S17, and S18, and Movie S8). The significantly higher Manders’ coefficient observed for CD20/RTX complexes and cholesterol‐rich nanodomains demonstrates their colocalization (Figure S18). Previous studies reported that CD20 molecules are homogeneously distributed in the membrane before RTX binding [43, 72]. Therefore, our observations are consistent with RTX‐associated nanoscale reorganization of CD20 and colocalization with cholesterol‐rich membrane domains (Figure S18). However, because EGFP‐D4 binding itself induced polarization in a subset of B cells (Figure S16), probe‐mediated effects may contribute to the observed degree of colocalization and should therefore be considered when interpreting these findings.

In contrast to RTX binding, binding of a monoclonal anti‐CD45 antibody did not induce polarization of CD45 on B cells (Figure 4k). However, B cell polarization was still visible in some cases most likely induced by EGFP‐D4 binding (Figures 4k and S16). In summary, ExM with EGFP‐D4 reveals the spatial association of CD20/RTX complexes with cholesterol‐rich membrane domains on the B cell membrane, which are preferentially localized on microvilli‐like protrusions (Figures 3, 4, and S11).

While RTX binding to CD20 has been studied in detail [30, 42, 43], knowledge about the molecular operating mode of DARA binding to CD38 on multiple myeloma cells remains elusive. Like RTX, DARA is proposed to crosslink CD38 to induce the formation of Fc fragment platforms that facilitate C1q binding and generation of the membrane attack complex resulting in subsequent permeabilization of the cell membrane [30, 42, 43, 67]. Knowledge about the association of CD38 with lipid nanodomains is only sparsely available [73]. To visualize the membrane distribution, multiple myeloma cells (RPMI 8226 cells) were treated with a mixture of CF568‐labeled DARA and EGFP‐D4, fixed and permeabilized, followed by expansion. Airyscan images of expanded MM cells showed pronounced colocalization of CD38/DARA complexes and cholesterol‐rich membrane domains on the cell surface (Figure 5a–e). However, both cholesterol‐rich membrane domains and CD38/DARA complexes are more homogenously distributed in the membrane of MM cells and less accumulated on microvilli than observed for CD20/RTX complexes and cholesterol‐rich membrane domains on B cells (Figures 4, 5, and Movie S9). In addition, multiple myeloma cells do show a much less pronounced polarization upon DARA and EGFP‐D4 binding (Figure 5f). For the DARA and EGFP‐D4 signals, we determined a Manders’ colocalization coefficient of 0.281 ± 0.096 (Figure 5g). Relative to CD45 as a non‐raft‐associated membrane reference, this value indicates a higher degree of association of CD38/DARA complexes with cholesterol‐rich membrane domains in multiple myeloma cells, albeit to a lower degree than observed for CD20/RTX complexes (Figure S18).

**FIGURE 5:**
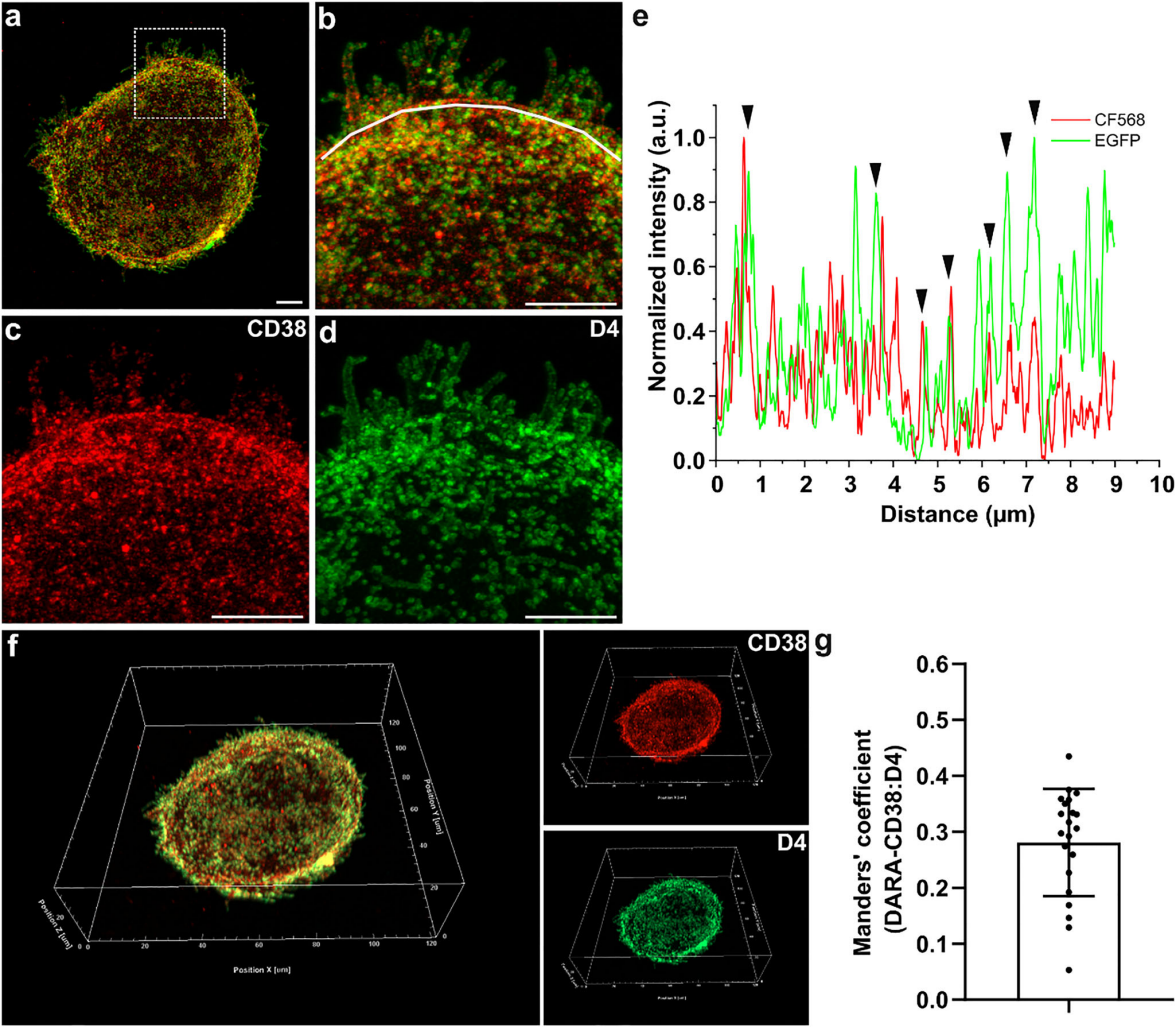
DARA binding to CD38 on MM cells induces redistribution of CD38/DARA complexes into cholesterol‐rich membrane domains. Multiple myeloma cells (RPMI 8226 cells) were labeled with EGFP‐D4 and CF568‐conjugated DARA for 15 min, fixed, permeabilized, and expanded. (a–d) Airyscan super‐resolution images of cholesterol‐rich membrane domains and CD38 (a) and zoom‐in view of the boxed region (b–d) after expansion. Cholesterol‐rich membrane domains (EGFP‐D4) are shown in green and DARA‐stained CD38 is in red. (e) Normalized fluorescence intensity profiles along the white line in (b), highlighting the colocalization of CD38/DARA and cholesterol‐rich membrane domains. The distance corresponds to the pre‐expansion dimension, adjusted according to the expansion factor. Black arrows indicate representative regions where CD38 and D4 signals colocalize. (f) Corresponding 3D image of cholesterol‐rich membrane domains and CD38. (g) Analysis of whole cell data yields a colocalization coefficient of DARA and cholesterol‐rich membrane domains on MM cells of 0.281 ± 0.096 (*n* = 21 cells). “DARA‐CD38:D4” represents the fraction of DARA‐CD38 signal overlapping with EGFP‐D4. Representative images were selected from *n* = 3 independent samples. Scale bars, 10 µm (a–d).

## 3 Conclusion

Immunotherapies with therapeutic antibodies (mAbs) have dramatically improved outcomes for many patients, offering durable responses where few options previously existed. Beyond cancer, therapeutic antibodies play a crucial role in treating autoimmune diseases, chronic inflammatory conditions, and infectious diseases by selectively restoring balance to the immune system or neutralizing harmful targets [28, 29, 30]. However, despite their important roles, molecular mechanisms of the interaction of therapeutic antibodies with membrane receptors are only poorly understood. Here RTX, the first approved mAb for the treatment of B cell‐related malignancies, remains an exception. Several studies showed that RTX binding to CD20 crosslinks CD20 homodimers and oligomers in the plasma membrane. Such receptor clustering generates larger platforms with high Fc fragment density that trigger C1q binding and complement‐dependent cytotoxicity [30, 42, 43, 67]. Although RTX serves as a reference for mAb operating modes, visualization of the binding‐induced association of CD20 with lipid nanodomains in the B cell membrane had so far not been achieved.

Here we used a fluorescent toxin probe (domain 4 (D4) of perfringolysin O) [50, 51, 52] that binds to cholesterol‐rich membrane domains, a structural element of lipid nanodomains, and can be linked covalently into hydrogels to visualize cholesterol‐rich domains on whole cells by the combination of ExM and Airyscan microscopy. Our results show that EGFP‐D4‐positive puncta exhibit an apparent average diameter of 64 ± 17 nm (mean ± s.d., *n* = 320) under the present imaging conditions and were frequently localized on actin‐supported membrane protrusions in COS‐7 cells. The apparent size range of these cholesterol‐rich membrane puncta is consistent with nanoscale membrane heterogeneity reported previously using complementary biophysical and super‐resolution microscopy approaches [17, 18, 20, 21]. The appearance of linear, chain‐like structures of cholesterol‐rich membrane domains on the basal membrane of cells can be explained by microvilli of insufficiently adhered cells sandwiched between the membrane and the coverslip [43]. In our study, we observed that cholesterol‐rich domains were frequently accumulated on microvilli‐like protrusions in COS‐7 and Raji B cells, consistent with previous studies showing that highly curved membrane protrusions such as microvilli or epithelial brush borders can exhibit enrichment of cholesterol‐ and sphingolipid‐rich membrane domains [74, 75]. In contrast, a seemingly lower accumulation of cholesterol‐rich domains was observed in RPMI 8226 cells, demonstrating that the abundance and spatial distribution of such membrane domains is cell‐type dependent and influenced by membrane composition, cytoskeletal organization, membrane curvature, and other cellular factors [2, 74, 75, 76].

Experiments with therapeutic mAbs RTX and DARA on B and MM cells, respectively, revealed partial colocalization of CD20/RTX and CD38/DARA complexes with cholesterol‐rich membrane domains visualized by EGFP‐D4. While CD20/RTX complexes are accumulated together with cholesterol‐rich membrane domains on microvilli of polarized B cells, CD38/DARA complexes and cholesterol‐rich membrane domains are more homogenously distributed throughout the cell membrane of MM cells. The extent to which receptor/mAb complexes are dragged toward microvilli is thus mainly controlled by the cell type. In the case of B cells, actin‐rich protrusions play a crucial role in early antigen recognition. Upon activation of B cells, they increase their cell surface area and create discrete nanodomains where receptors, signaling molecules, and lipids are locally enriched [61, 71]. In this light, the preferential association of CD20/RTX complexes with cholesterol‐rich membrane domains on microvilli‐like protrusions may reflect antibody‐induced reorganization of membrane receptors in B cells. Previous studies have suggested that local high Fc fragment concentrations are required to initiate immune effector functions, including antibody‐dependent cellular cytotoxicity (ADCC), antibody‐dependent cellular phagocytosis (ADCP), and complement‐dependent cytotoxicity (CDC) [30, 77]. Regarding complement activation efficiency of mAbs it makes sense to redistribute receptor/mAb complexes into small cholesterol‐rich membrane domains to increase the local Fc density and promote binding of hexa‐headed C1q. This might be of minor importance for mAbs like RTX which efficiently crosslinks oligomeric CD20 and forms larger Fc‐rich nanodomains [43], but it can significantly improve complement activation for mAbs/receptor pairs that exhibit a lower crosslinking efficiency, for example, if the receptor is expressed as monomer in the plasma membrane of tumor cells. In addition, particularly for those receptors that are expressed at low concentrations, accumulation of receptor/mAb complexes in cholesterol‐rich membrane domains can significantly improve C1q binding efficiency and thus the efficacy of immunotherapies.

In contrast to CD20/RTX complexes in B cells, the more homogeneous membrane distribution of CD38/DARA complexes in multiple myeloma cells demonstrates different membrane architecture and receptor organization. CD38 is a multifunctional ectoenzyme and surface receptor whose distribution and organization on the plasma membrane appears to be less dependent on clustering. While RTX crosslinks homodimeric CD20 in the plasma membrane of B cells inducing the formation of CD20/RTX nanodomains of different geometry with high Fc fragment density, binding of DARA to CD38 induces a weaker crosslinking efficiency. However, accumulation of CD38/DARA complexes in cholesterol‐rich nanodomains can result in locally higher Fc fragment density that can initiate CDC, ADCC, and ADCP. In addition, DARA can exert its therapeutic activity through other immunomodulatory mechanisms [44, 73]. Therefore, a more homogeneous membrane distribution of CD38/DARA complexes may facilitate interactions with immune effector components across larger regions on the tumor cell surface. Overall, our results show that 4x ExM serves as a powerful method for multicolor visualization and mapping of the interaction of mAbs with lipid nanodomains.

## Author Contributions

**Gang Wen**: conceptualization, investigation, writing – original draft, methodology, validation, visualization. **Nicole Seifert**: methodology, writing – review and editing. **Patrick Eiring**: writing – review and editing, methodology. **Johanna Meyer**: methodology, writing – review and editing. **Hannes Neuweiler**: methodology, writing – review and editing. **Julia Weingart**: methodology, writing – review and editing. **Ignacio Vega‐vásquez**: methodology, writing – review and editing. **Sören Doose**: software, writing – review and editing. **Philip Kollmannsberger**: software, writing – review and editing. **Markus Sauer**: conceptualization, funding acquisition, writing – original draft, project administration, supervision, writing – review and editing.

## Funding

This work was supported by the European Research Council (ERC) under the European Union's Horizon 2020 research and innovation programme (grant agreement No. 835102), and Research Fund from Soochow University (No. NH12800826).

## Conflicts of Interest

The authors declare no conlicts of interest.

## Supporting information




**Supporting File 1**: anie74279‐sup‐0001‐SuppMat.zip.

**Supporting File 2**: anie74279‐sup‐0002‐SuppMat.pdf.

**Supporting File 3**: anie74279‐sup‐0003‐SuppMat.zip.

## Data Availability

The data that support the findings of this study are available from the corresponding author upon reasonable request.
