# Supplementary material for: Expansion Microscopy Reveals the Spatial Association of Therapeutic Antibody–Receptor Complexes With Cholesterol‐Rich Membrane Domains
Source: Angew Chem Int Ed Engl. 2026 Aug 24;65(42):e1206161. doi: 10.1002/anie.1206161 (PMC13653619; doi:10.1002/anie.1206161)
Supplement: Supplementary file 1 [file ANIE-65-e1206161-s002.pdf]

Supporting Information

Expansion Microscopy Reveals the Spatial Association of Therapeutic Antibody–Receptor Complexes with Cholesterol-Rich Membrane Domains

Gang Wen[1,2]*, Nicole Seifert[2], Patrick Eiring[2], Johanna Meyer[2], Hannes Neuweiler[2], Julia Weingart[2], Ignacio Vega-Vásquez[2], Sören Doose[2], Philip Kollmannsberger[2], Markus Sauer[2,3]*

[1]State Key Laboratory of Radiation Medicine and Protection, School of Radiation Medicine and Protection, Collaborative Innovation Center of Radiological Medicine of Jiangsu Higher Education Institutions, Soochow University, Suzhou, 215123, P. R. China

[2]Department of Biotechnology and Biophysics, Biocenter, University of Würzburg, Am Hubland, 97074 Würzburg, Germany

[3]Rudolf Virchow Center, Research Center for Integrative and Translational Bioimaging, University of Würzburg, Josef-Schneider-Str. 2, 97080 Würzburg, Germany

Corresponding authors: G.W. (gang.wen@uni-wuerzburg.de) and M.S. (m.sauer@uni-wuerzburg.de)

MATERIALS AND METHODS

Cell culture. The complete growth medium containing DMEM/HAM's F12 medium, fetal bovine serum (FBS, 10%), penicillin (100 U/mL) and streptomycin (0.1 mg/mL) was used to culture COS-7 cells in a humidified incubator at 37 °C with 5% CO_2. COS-7 cells were plated in 4-well plates containing a 12 mm coverslip (high precision, No. 1.5H) at a density of $7×10^4$ cells per well. After seeding, cells were grown overnight before staining. Raji (ATCC-CCL-86, Batch: 70015320) and RPMI-8226 cells (DSMZ, #ACC 402) were cultivated in RPMI-1640-media (Sigma, R8758) with 10% FCS (Sigma, F7524), 100 U/mL Penicillin and 0.1 mg/mL Streptomycin (Sigma, P4333) at 37 °C and 5% CO_2 at a maximum density of $1x10^6$ cells/mL in standard T25 culture flasks (Sarstedt, # 83.3910.502).

Recombinant synthesis of EGFP-D4 probes. pET28 vectors containing the gene of EGFP-D4 as N-terminal His_6-tagged fusion constructs were received from RIKEN BioResource Research Center (Ibaraki, Japan). Plasmids were transformed into heat shock competent *Escherichia coli* C41 (DE3) E. coli bacterial cells. Liquid cultures in 2xTY medium containing 50 µg/mL kanamycin were grown at 37 °C until an optical density (600 nm) of 0.8 was reached. Over-expression was induced by adding 1 mM IPTG. The temperature was reduced to 30 °C and liquid cultures were further incubated overnight. Cells were harvested by centrifugation at 4000 rpm for 15 minutes and resuspended in 50 mM phosphate, pH 8.0, containing 10 mM imidazole. Cells were lysed through ultrasonication and application of lysozyme. Cell lysates were clarified through centrifugation at 15,000 rpm for 60 minutes, followed by filtration through a 0.2 µm syringe filter. Clarified cell lysates were applied to Ni-NTA affinity chromatography using Ni Sepharose excel resin (Cytiva). His_6-tagged protein constructs were eluted using 300 mM imidazole, pH 8.0. Purified proteins were concentrated using centrifugal concentrators (Sartorius Vivaspin 20, 10 kDa MWCO) and the protein concentration was measured via UV VIS analysis. The toxin probes were stored as 50% glycerol solution at −20 °C.

Anti-receptor antibody conjugation with organic dyes. Conjugation of daratumumab with CF568-NHS was performed following the protocol below. Briefly, 50 µg of antibodies was buffer-exchanged into 100 mM sodium bicarbonate buffer (pH 8.5) using 0.5 mL spin desalting columns (40K MWCO, Thermofisher, #87766). To achieve an average Degree of Labeling (DOL) of 2-3, CF568-NHS (5 mol equivalents) was added to the reaction solution. The conjugated antibody was purified and washed using additional spin desalting columns to remove unbound dye. DOL and the antibody concentration were calculated according to the following formula.

$$Protein\,concentration\,(M) = \frac{A_{280} - (A_{dye}\,x\,CF)}{\varepsilon^{protein}}\,x\,dilution\,factor$$

$$Degree\,of\,labeling\,(DOL) = \frac{A_{max}}{\varepsilon^{dye}\,x\,protein\,concentration\,(M)}\,x\,dilution\,factor$$

Rituximab (Rituxan, Roche) and CD45 clone HI30 (Biolegend, 304002) were conjugated to CF568-NHS (Merck, #SCJ4600027). The buffer was exchanged into 200 mM sodium bicarbonate (Fisher Scientific, S/4240/60) using spin desalting columns (40 K MWCO, Fisher-Scientific GmbH, A57760). 100 µg antibody was incubated with 5 µg CF568-NHS at RT for 3 h to receive an average DOL of 3-5. With an additional spin column unbound dye was removed. DOL and the antibody concentration were calculated according to the abovementioned formula.

Cholesterol-rich membrane domain staining in COS-7 cells. Cultured cells were rinsed twice with ice-cold DMEM medium and incubated with 1.2 µM pET28/His6-EGFP-D4 (300 µL) in DMEM for 30 min at 4 °C for cholesterol-rich domain staining, unless otherwise noted. Next, cells were then quickly rinsed twice with ice-cold Dulbecco’s phosphate buffered saline (DPBS) and fixed with 3% PFA and 0.1% GA in ice-cold DPBS on ice for 5 min and then for 20 min at room temperature. After quenching with freshly prepared 0.1% sodium borohydride for 7 min, cells were washed three times with DPBS. Fixed cells were then permeabilized with 0.2% Triton X-100 for 12 min, washed three times with DPBS and stained with DAPI (1 µg/mL) for

2 min. Afterward, cells were washed three times again with DPBS.

Evaluation of probe binding specificity in cholesterol-depleted cells. For cholesterol depletion, cultured cells were fed with 20 mM methyl-β-cyclodextrin (MβCD) in the complete growth medium for 90 min. After lipid depletion, cells were quickly rinsed twice with DMEM and stained with EGFP-D4 probes as described previously.

Multi-color imaging in COS-7 cells. Cholesterol-rich domains were stained as mentioned above. For multi-color staining with antibodies, after fixation and permeabilization, cells were treated with freshly prepared blocking buffer (3% BSA in DPBS) for 20 min at room temperature. Cells were then stained with 5 μg/mL primary anti-clathrin antibodies (Rabbit, ab21679), primary anti-LAMP1 antibodies (Mouse, ab25630, H4A3), or primary anti-Rab 5A antibodies (Mouse, 2E8B11) in staining buffer (3% BSA in DPBS) for 1 h at room temperature, unless otherwise noted, followed by washing three times with DPBS for 5 min. Samples were then incubated with 20 μg/mL fluorescently labeled secondary antibodies as indicated for 1 h (CF568-modified goat anti-Rabbit $F(ab)_2$ IgG, ATTO 643-modified goat anti-Mouse IgG, or CF568-modified goat anti-Mouse IgG, respectively). Cells were then rinsed twice with DPBS, washed twice with 1% BSA in DPBS and washed once with DPBS. Stained cells were then stained with DAPI (1 μg/mL) for 2 min and washed three times with DPBS before imaging. For multi-color staining with multifunctional phalloidin linkers, after fixation and permeabilization, cells were stained with 0.5 μM "Actin ExM" kit (fluorophore 561) in DPBS at RT for 1 h and washed three times with DPBS. Next, cells were stained with DAPI (1 μg/mL) for 2 min and washed three times with DPBS before imaging.

Multicolor staining in Raji cells or multiple myeloma cells. To coat Raji cells or multiple myeloma cells on coverslips, 12 mm coverslips in 4-well plates were incubated with 0.1 mg/mL poly-D-lysine at room temperature for 1 h, rinsed once with water and then let it air-dry prior to use. Cultured Raji cells were grown on poly-D-lysine-coated coverslips at a density of 5×10^5 cells per well for 1 h. Next, cells were rinsed twice with pre-warmed DPBS and incubated with a mixture of 1.2 μM pET28/His6-EGFP-D4 and 5 μg/mL CF568-conjugated RTX (anti-CD20 antibodies) or 5 μg/mL CF568-conjugated

anti-CD45 antibodies in pre-warmed DPBS (300 μL) for 15 min at 37 °C. Next, cells were quickly rinsed once with pre-warmed DPBS and fixed with 3% PFA and 0.1% GA in pre-warmed DPBS at room temperature for 15 min, followed by the quenching and permeabilization steps as mentioned above. Cultured multiple myeloma cells (RPMI 8226 cells) were grown on poly-D-lysine-coated coverslips at a density of 4×10^5 cells per well for 1 h. Cells were rinsed twice with pre-warmed DPBS and incubated with a mixture of 1.2 μM pET28/His6-EGFP-D4 and 5 μg/mL CF568-conjugated anti-CD38 antibodies in pre-warmed DPBS (300 μL) for 15 min at 37 °C. Next, cells were quickly rinsed once with pre-warmed DPBS and fixed with 3% PFA and 0.1% GA in pre-warmed DPBS at room temperature for 15 min, followed by the quenching and permeabilization steps as mentioned above.

Expansion microscopy. ExM was performed as previously reported (*35*). The monomer solution of 4x ExM was prepared with 2 M NaCl, 86 mg/mL sodium acrylate, 25 mg/mL acrylamide, and 1.5 mg/mL N,N'-methylenebis(acrylamide) in 1x PBS and stocked at -20 °C before use. The samples were treated with AcX (0.1 mg/mL) in PBS at room temperature for 90 min, followed by washing three times with DPBS before gelation. For 4x ExM, samples were first rinsed with 50 μL freshly prepared gelation solution (4x ExM monomer solution supplemented with 0.01% 4-hydroxy-TEMPO, 0.2% tetramethylenediamine (TEMED, w/w), and 0.2% ammonium persulfate (APS, w/w)) and then the polymerization was performed on the gelation chamber with another 30 μL gelation solution on ice for 5 min, followed by room temperature for 90 min. Next, 8 U/mL proteinase K (New England Biolabs) in digestion buffer containing 50 mM Tris (pH 8.0), 1 mM EDTA, 0.5% TritonX-100, and 0.8 M guanidine HCl was applied to digest gels at room temperature for 12 h. The samples were then rinsed once with DPBS to remove proteinase K, re-stained with DAPI (3 μg/mL) for 5 min and washed three times with DPBS. Afterward, digested samples were immersed in deionized water for expansion. Deionized water was replaced every 15 min with fresh water and this process was repeated four times until the gels reached full expansion.

Stabilization of expanded gels. To stabilize the gels, expanded samples were mounted on silanized one-well Cellvis chambers (Product #: C1-1.5H-N) prior to imaging. In brief, the chamber was washed with deionized water for 15 min and 1 M KOH aqueous solution for another 15 min. After rinsing three times with deionized water, the chamber was washed with deionized water for 15 min and washed with ethanol for 15 min. Chambers were then air-dried. Afterward, the chamber was incubated with 2 mL silanization solution (Recipe: 9 mL ethanol, 0.5 mL deionized water, 0.5 mL acetic acid, and 10 μL 3-aminopropyltriethoxysilane) for 15 min. The chamber was air-dried and rinsed once with ethanol and let it air-dry again before using.

Fluorescence microscopy and image analysis. Confocal images and Airyscan SR images were acquired using the LSM 900 equipped with Airyscan 2 (Zeiss) system. Confocal images were performed on a C-Apochromat 40x/1.2 numerical aperture (NA) water-immersion objective. Airyscan SR images were performed on a Pln Apo 63x/1.4 NA oil-immersion objective or a C-Apochromat 40x/1.2 NA water-immersion objective. This system was operated through the ZEN 2 blue software (version 3.5, Zeiss). Samples labelled with EGFP and various organic fluorophores were excited and emitted using system's integrated preset settings. Airyscan SR images were processed using the automatic parameters of 3D Airyscan processing mode. After image acquisition, all images were handled with ImageJ. The same features of cellular structures before and after expansion were compared to calculate the expansion factor achieved. Line profiles of expanded cellular structures were analysed using the "Plot Profile" function in ImageJ and subsequently rescaled according to the corresponding expansion factor. Puncta diameters were determined from fluorescence intensity line profiles using ImageJ, where full width at half maximum (FWHM) values were extracted from Gaussian-fitted intensity distributions along defined line scans across individual puncta. Colocalization analysis was performed using the "JACoP" plugin in ImageJ on z-stack images of the plasma membrane region. Intensity thresholds were set based on the nanoscale imaging signal and detailed parameters were included in the supplementary material. The cell-to-cell variability of Manders' values was accounted

for by analyzing multiple independent cells with statistical reporting as mean ± s.d.. Cell polarization was quantified by measuring fluorescence intensities in polarized membrane regions and across the entire cell. A fluorescence ratio was calculated as the fluorescence intensity within the indicated membrane region divided by the total cellular fluorescence intensity.

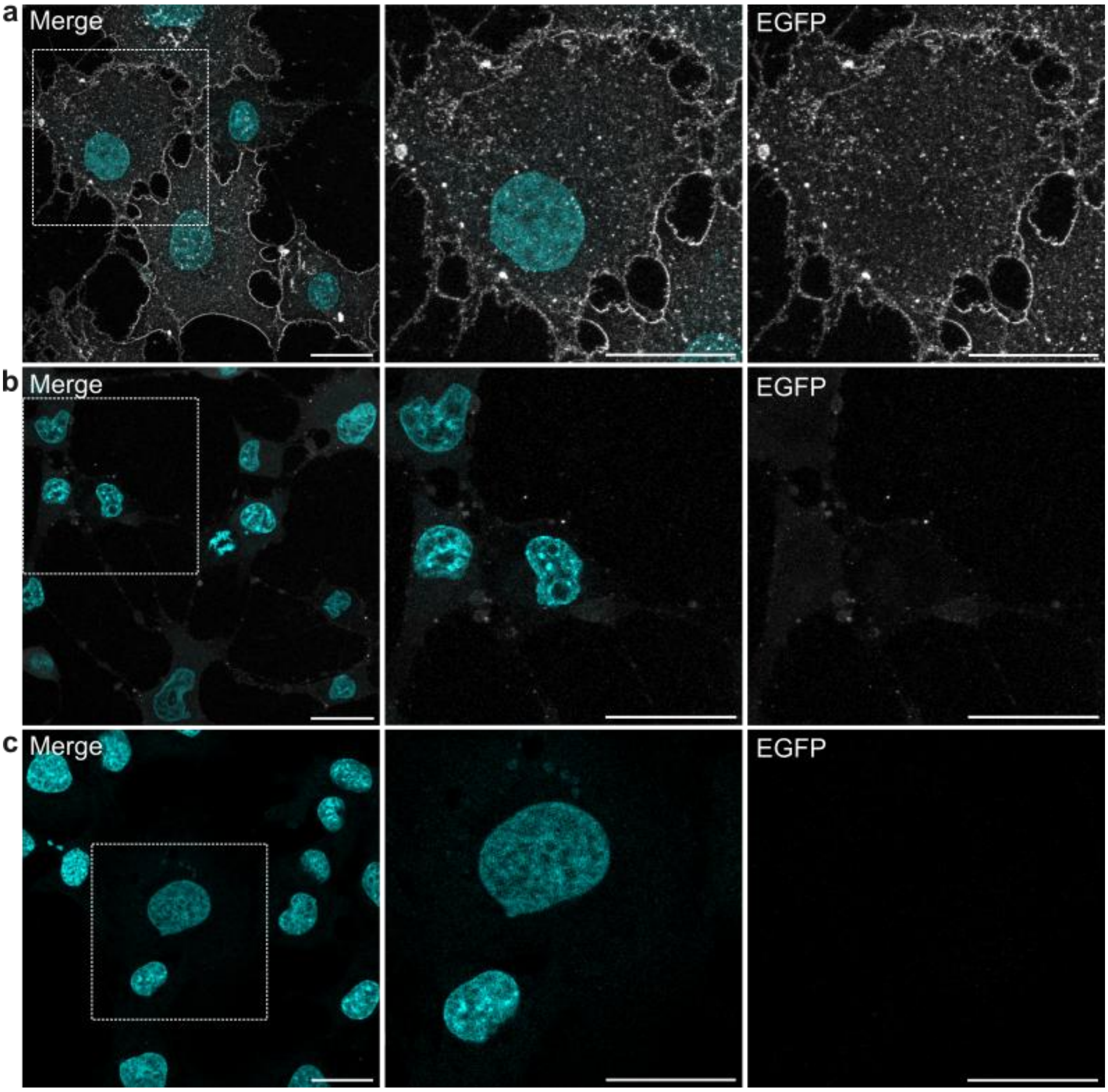

Figure S1. Characterization of EGFP-D4 for cholesterol-enriched domain staining. **a**, Confocal fluorescence image of cholesterol-rich domains visualized with EGFP-D4. COS-7 cells were labeled with EGFP-D4, fixed and permeabilized. DNA was stained with DAPI. **b**, Confocal fluorescence image of cholesterol-rich domains visualized with EGFP-D4 after MβCD treatment. COS-7 cells were first incubated with 20 mM MβCD in the complete growth medium for 90 min, followed by labeling with EGFP-D4, fixation and permeabilization. DNA was stained with DAPI. **c**, Confocal fluorescence image of cholesterol-rich domains in control experiments, where no EGFP-D4 was applied to COS-7 cells. All images were acquired with the same parameters. Representative images were selected from n= 3 independent samples. Scale bars, 30 μm.

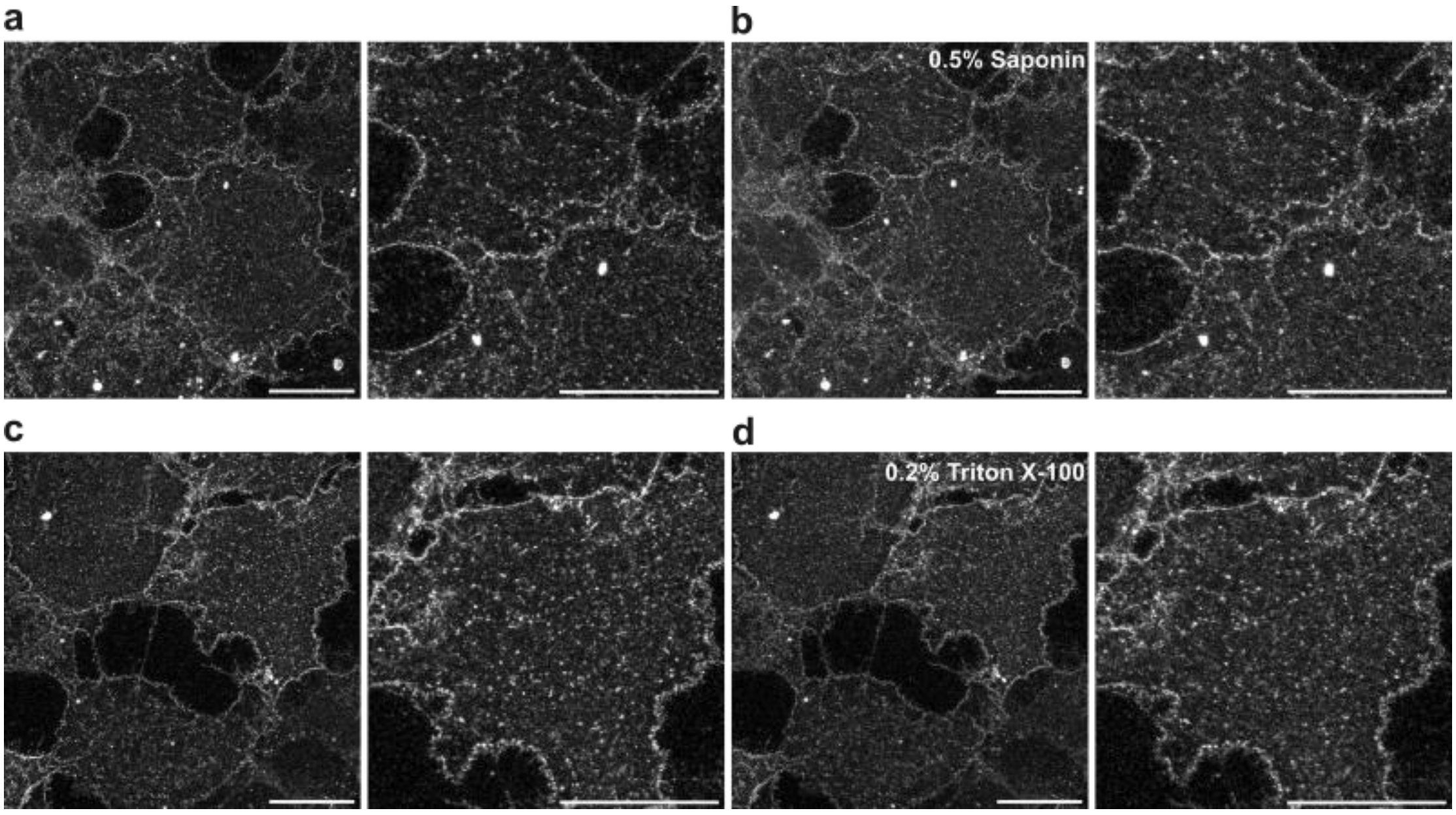

Figure S2. Fixability of EGFP-D4 probes. a-**b**, Comparison of fluorescence signal of cholesterol-rich membrane domains before (**a**) and after permeabilization with 0.5% saponin (**b**). **c**-**d**, Comparison of fluorescence signal of cholesterol-rich membrane domains before (**c**) and after permeabilization with 0.2% Triton X-100 (**d**). Representative images were selected from n= 3 independent samples. Scale bars, 30 μm.

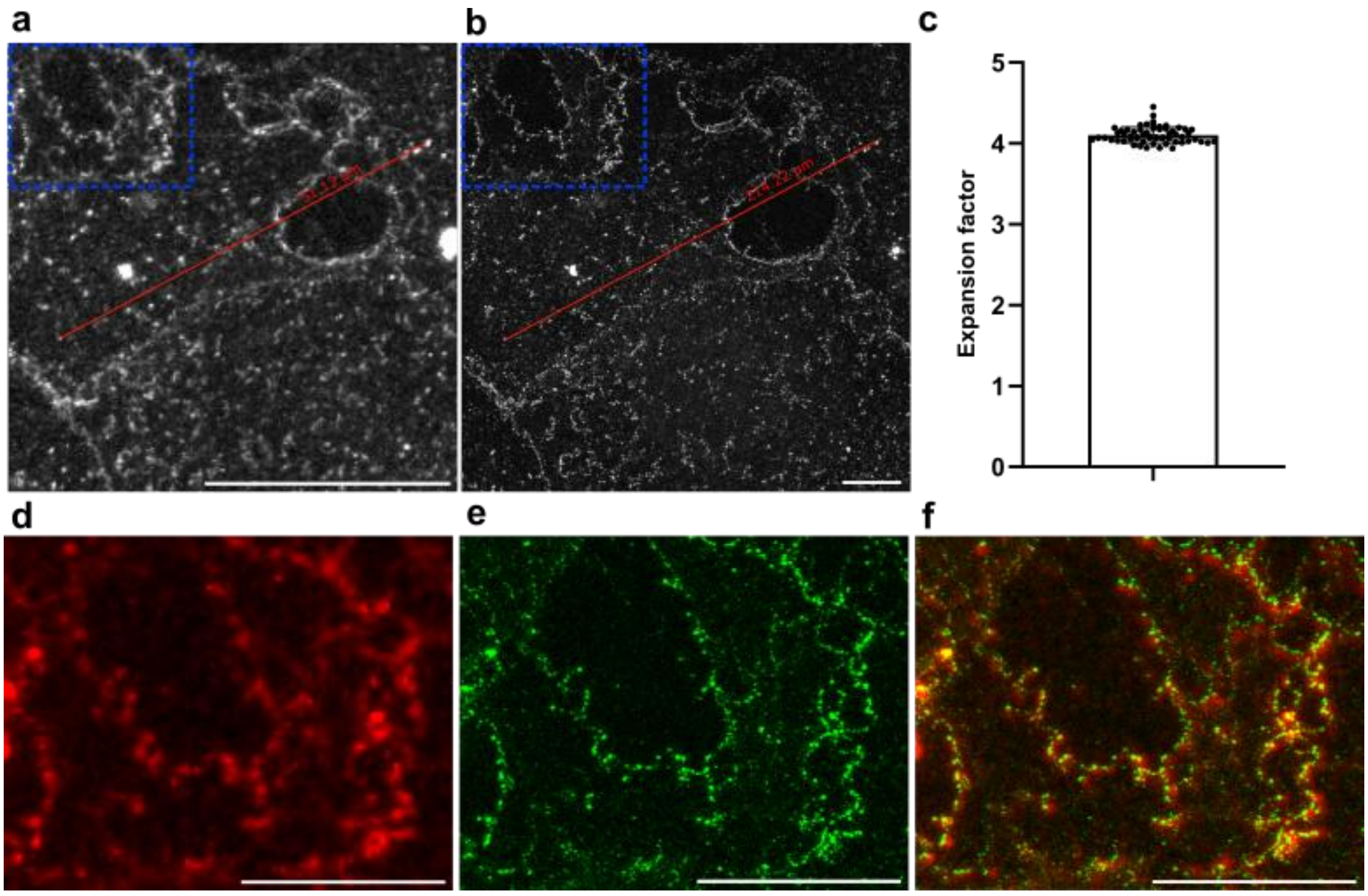

Figure S3. Comparison of the same cholesterol-rich membrane regions before and after expansion in 4x ExM. a, Representative image of cholesterol-rich domains before expansion. **b**, Post-expansion image of the same cells. **c**, Distribution of expansion factor determined by comparing cholesterol-rich membrane domains before and after expansion in the same cell. After 4-fold ExM we determined an expansion factor of 4.10 ± 0.10 (mean ± standard deviation, n= 60 from 30 cells in three independent samples). **d**,**e**, Magnified views of the boxed regions in panels **a** and **b**, respectively. **f**, Merged image of panels **d** and **e**. The scale bar of panel **e** is corresponding to pre-expansion. Scale bars, 30 μm (**a**,**b**) and 10 μm (**d**-**f**).

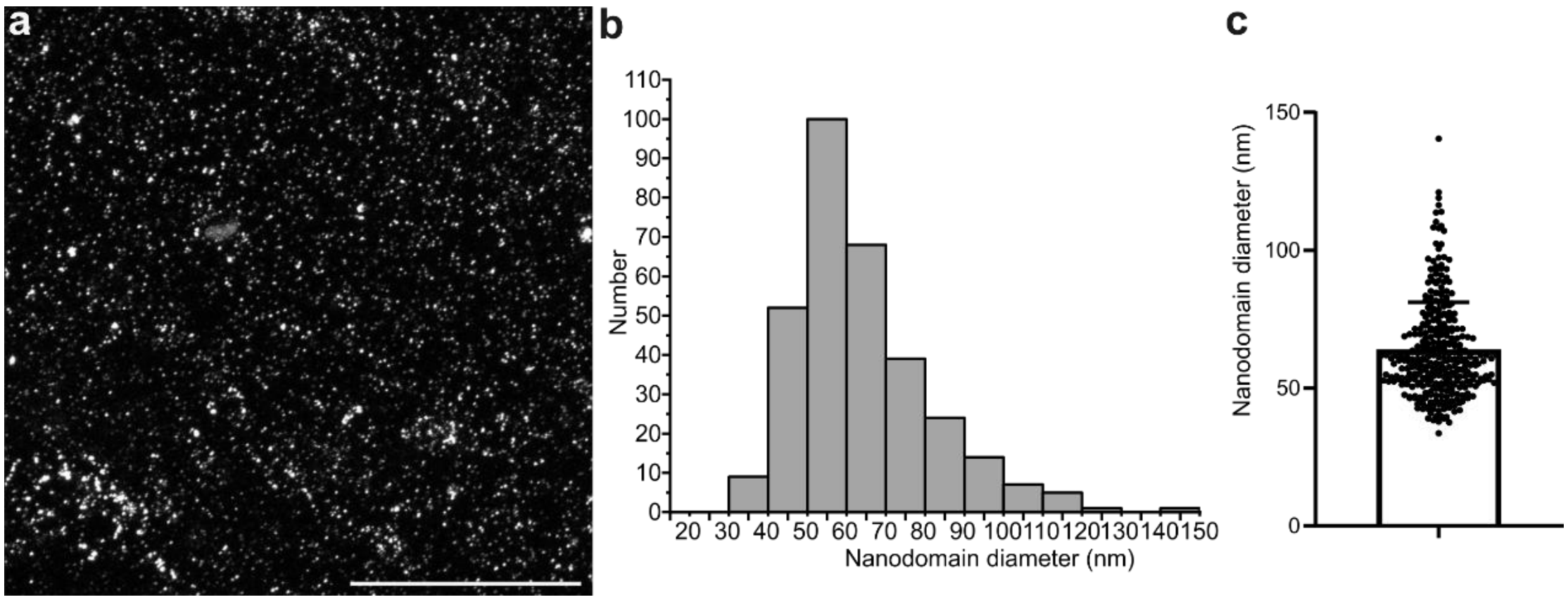

Figure S4. Size analysis of cholesterol-rich membrane domains using 4x ExM-airyscan microscopy. **a**, Representative image of cholesterol-rich domains after expansion using airyscan microscopy. **b**,**c**, Distribution of sizes of cholesterol-rich domains. 320 domains were analyzed. Representative images were selected from n= 3 independent samples. Scale bar, 30 μm.

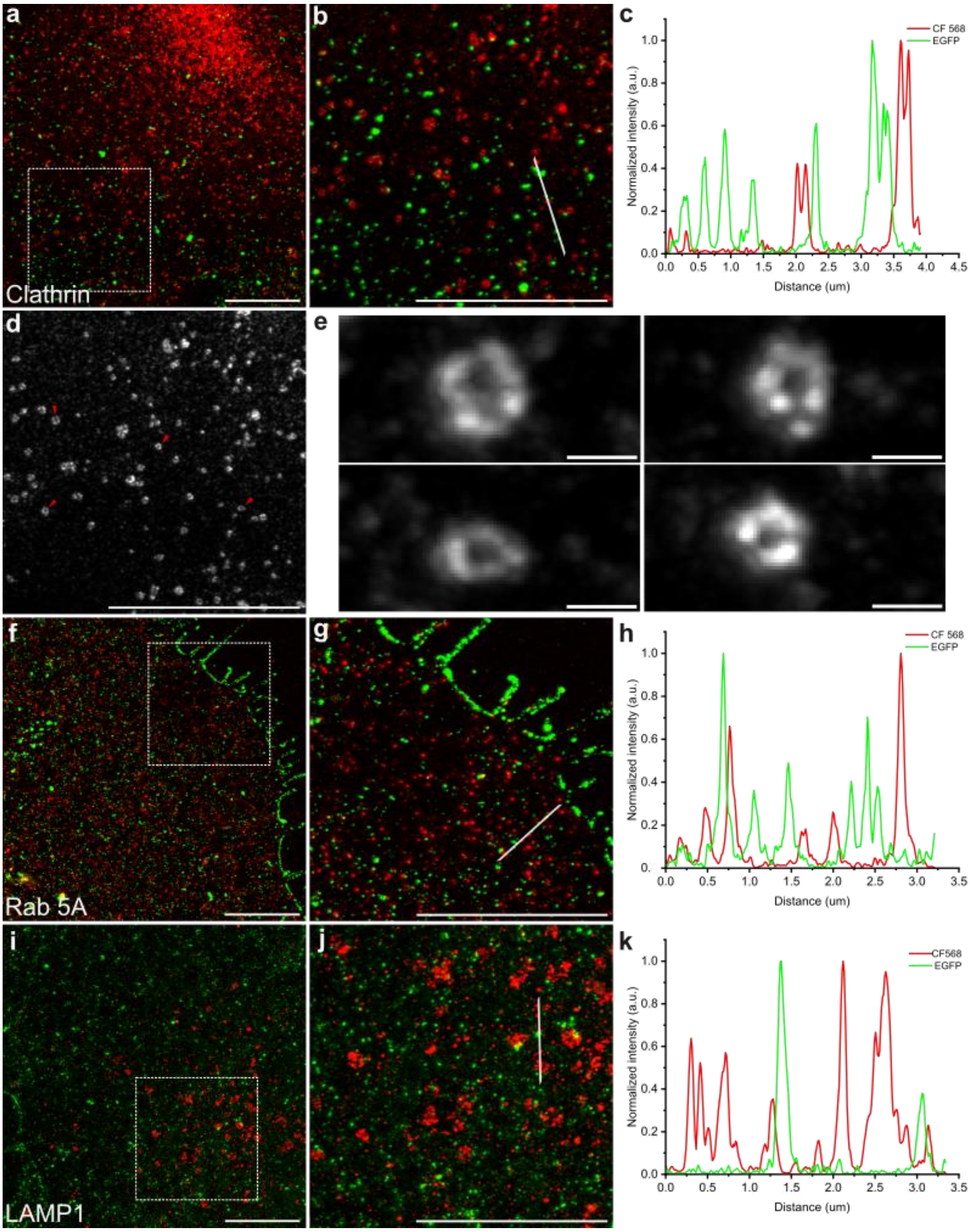

Figure S5. Imaging of cholesterol-rich membrane domains and vesicle markers using 4x ExM-airyscan microscopy. COS-7 cells were stained with EGFP-D4 at 4 °C, fixed, permeabilized, stained with antibodies against vesicles and expanded using the procedure of 4x ExM. **a**,**b**, Airyscan super-resolution images of cholesterol-rich membrane domains (EGFP, green) and clathrin-coated pits (CF568, red) acquired with a 40x water objective. **c**, Normalized fluorescence intensity profiles along the white lines in **b**. **d**, The separated channel of clathrin-coated pits in panel **b**. **e**, Individual clathrin-coated pits, showing the ring-like structure. **f**,**g**, Airyscan super-resolution images of cholesterol-rich membrane domains and early endosomes (**f**) and zoom-in view of the highlighted region (**g**). **h**, Normalized fluorescence intensity profiles along

the white lines in **g**. **i**,**j**, Airyscan super-resolution images of cholesterol-rich membrane domains and LAMP1-positive compartments (**i**) and zoom-in view of the highlighted region (**j**). **k**, Normalized fluorescence intensity profiles along the white lines in j. In **c**,**h**,**k**, the distance corresponds to the pre-expansion dimension, adjusted according to the expansion factor. Representative images were selected from n= 3 independent samples. Scale bars, 30 μm (**a**,**b**,**d**,**f**,**g**,**i**,**j**) and 1 μm (**e**).

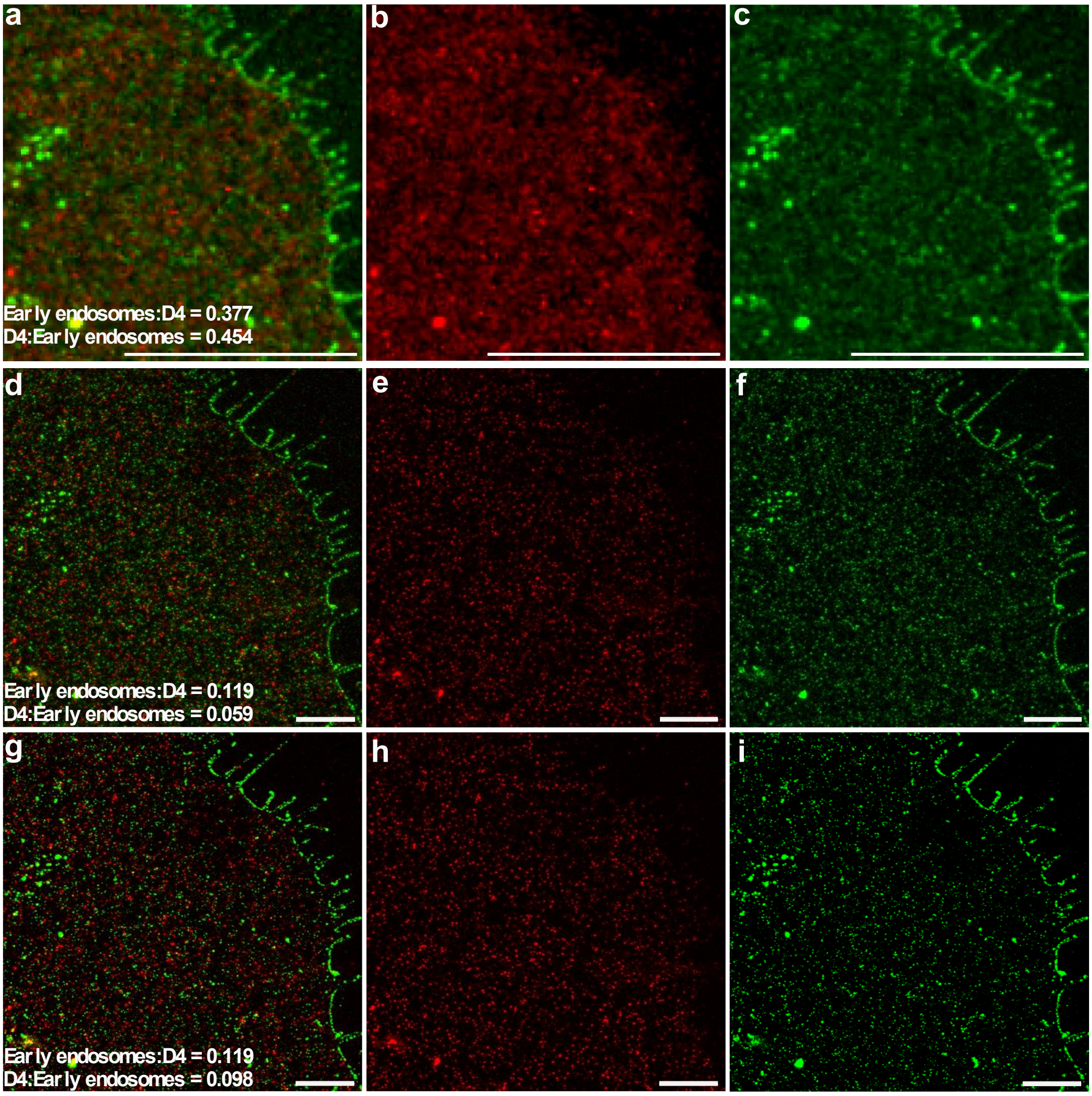

Figure S6. Multi-color images of cholesterol-rich domains and immunostained early endosomes in COS-7 cells using 4x ExM. a, Pre-expansion confocal fluorescence image of cholesterol-rich domains visualized with EGFP-D4 (green) and early endosomes (CF568, red). **b**,**c**, Red and green channel in **a**. **d**, Post-expansion confocal fluorescence image of cholesterol-rich domains and early endosomes obtained after 4x ExM in the same cell. **e**,**f**, Red and green channel in **d**. **g**, Post-expansion Airyscan SR image of cholesterol-rich domains visualized with EGFP-D4 (green) and early endosomes (CF568, red). **h**,**i**, The red and green channel in g. Colocalization coefficients of early endosomes and cholesterol-rich domains stained with D4 were analyzed using Manders' coefficients. "Early endosomes:D4" represents the fraction of early endosomes overlapping with D4; "D4:Early endosomes" represents the fraction of D4 overlapping with early endosomes. Representative images were selected from n= 3 independent samples. Scale bars, 20 μm.

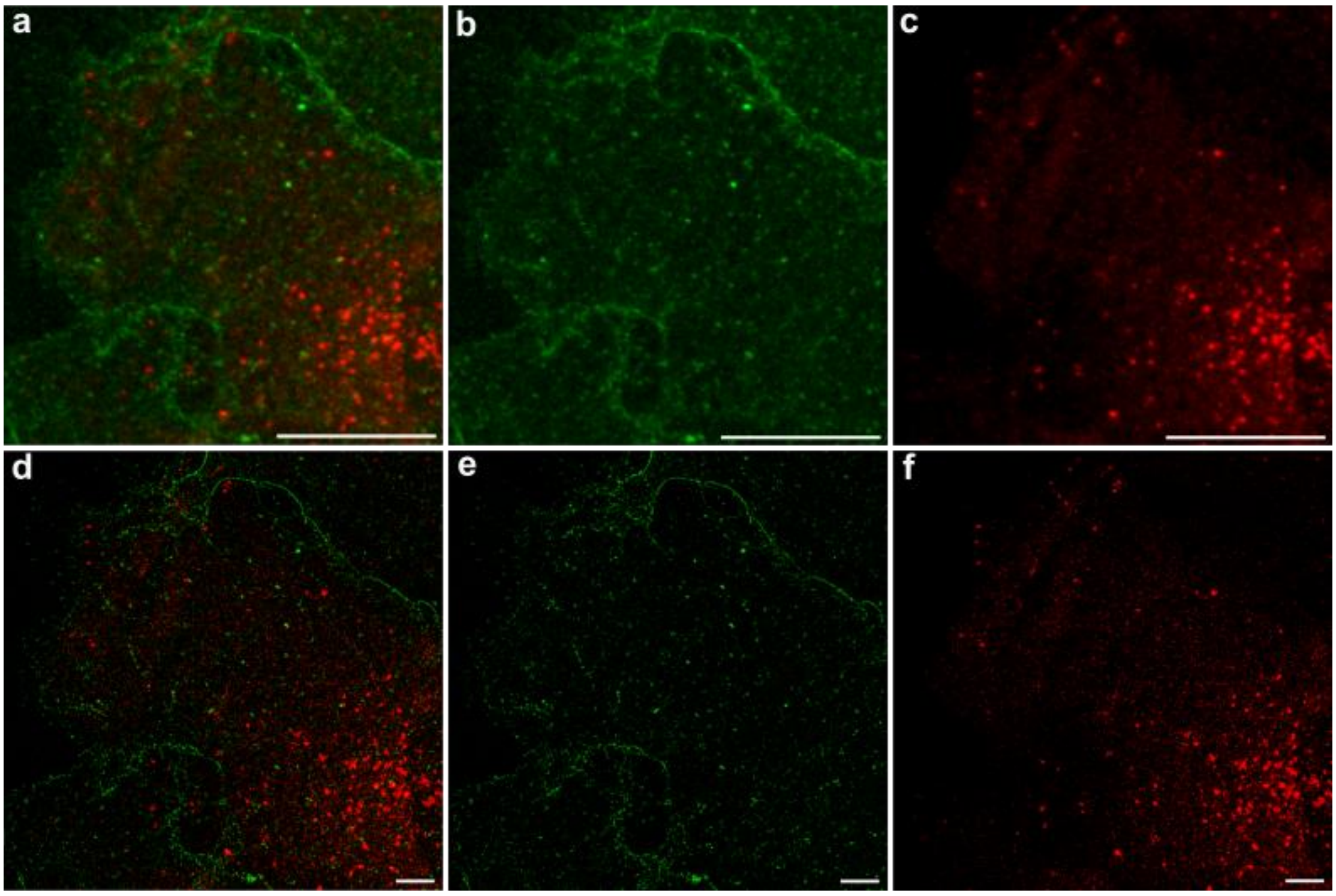

Figure S7. Multicolor images of cholesterol-rich domains and immunostained LAMP1-positive compartments in COS-7 cells using 4x ExM. a, Pre-expansion confocal fluorescence image of cholesterol-rich domains visualized with EGFP-D4 (green) and LAMP1-positive compartments (CF568, red). **b**,**c**, The green and red channel in **a**. **d**, Post-expansion confocal fluorescence image of cholesterol-rich domains and LAMP1-positive compartments obtained after 4x ExM in the same cell. **e**,**f**, The green and red channel in **d**. Representative images were selected from n= 3 independent samples. Scale bars, 20 μm.

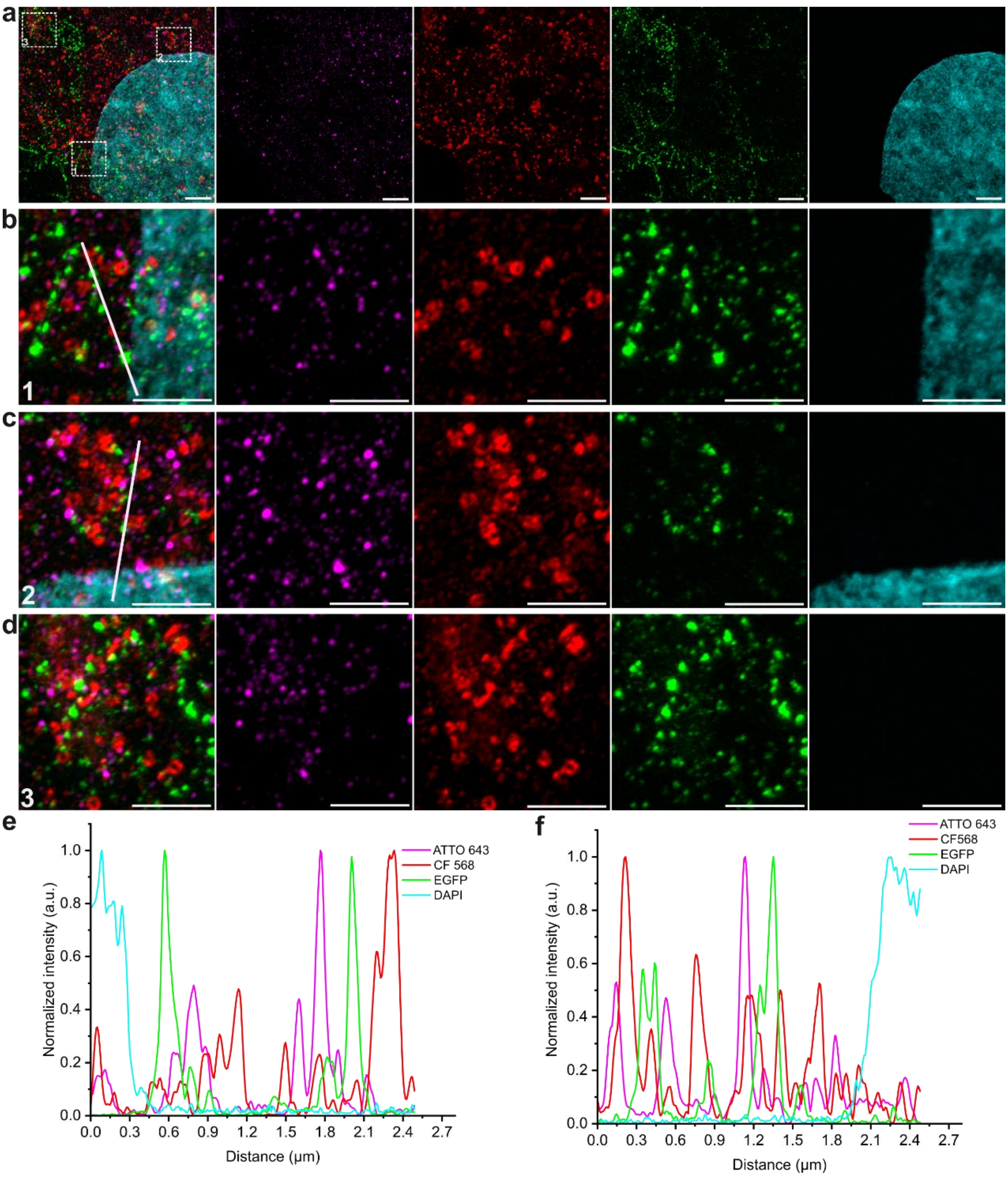

Figure S8. Multicolor imaging of cholesterol-rich membrane domains, clathrin-coated pits, early endosomes and nuclei using 4x ExM-airyscan microscopy. COS-7 cells were stained with EGFP-D4 at 4 °C, fixed, permeabilized, stained with antibodies against vesicles and expanded using the procedure of 4x ExM. **a**, Airyscan super-resolution images of cholesterol-rich membrane domains (EGFP-D4, green), clathrin-coated pits (CF568, red), early endosomes (ATTO 643, magenta) and nuclei (DAPI, cyan) acquired on a 63x oil objective. **b-d**, Zoom-in views of the highlighted region in **a**. **e**,**f** Normalized fluorescence intensity profiles along the white lines in **b** and **c**, respectively. In **e**,**f**, the distance corresponds to the pre-expansion dimension, adjusted according to the expansion factor. Representative images were selected from n= 2 independent samples. Scale bars, 10 μm (**a**) and 5 μm (**b-d**).

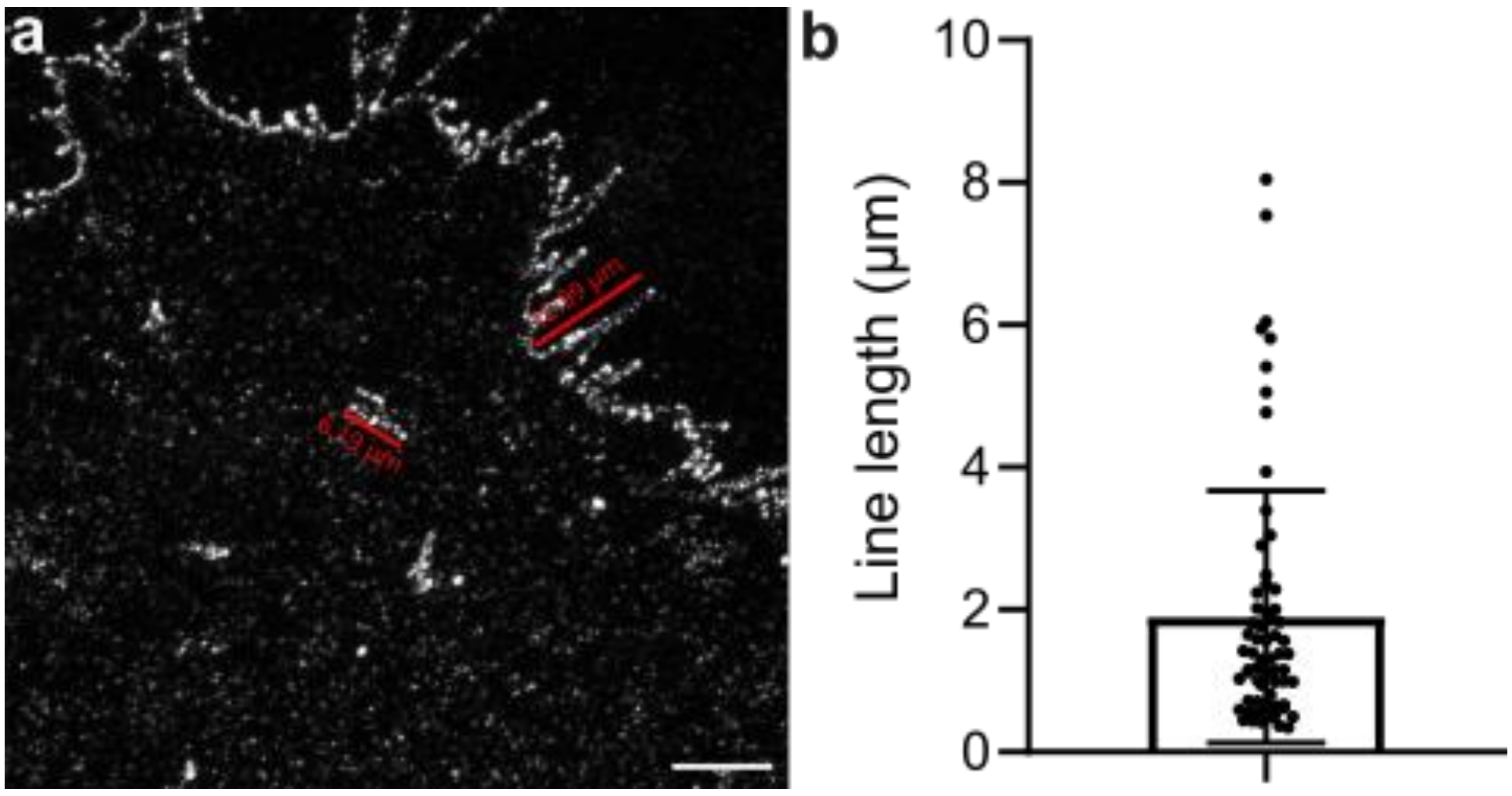

Figure S9. Quantification of the length of membrane protrusions obtained using Airyscan microscopy-4x ExM. **a**, Representative post-expansion image of cholesterol-rich domains after 4x ExM. **b**, Distribution of the length of microvilli-like protrusions. Representative images were from three independent samples, yielding a line length of 0.34 -8.0 μm (n= 66 from three independent samples). The length corresponds to the pre-expansion dimension, adjusted according to the expansion factor. Scale bar, 10 μm.

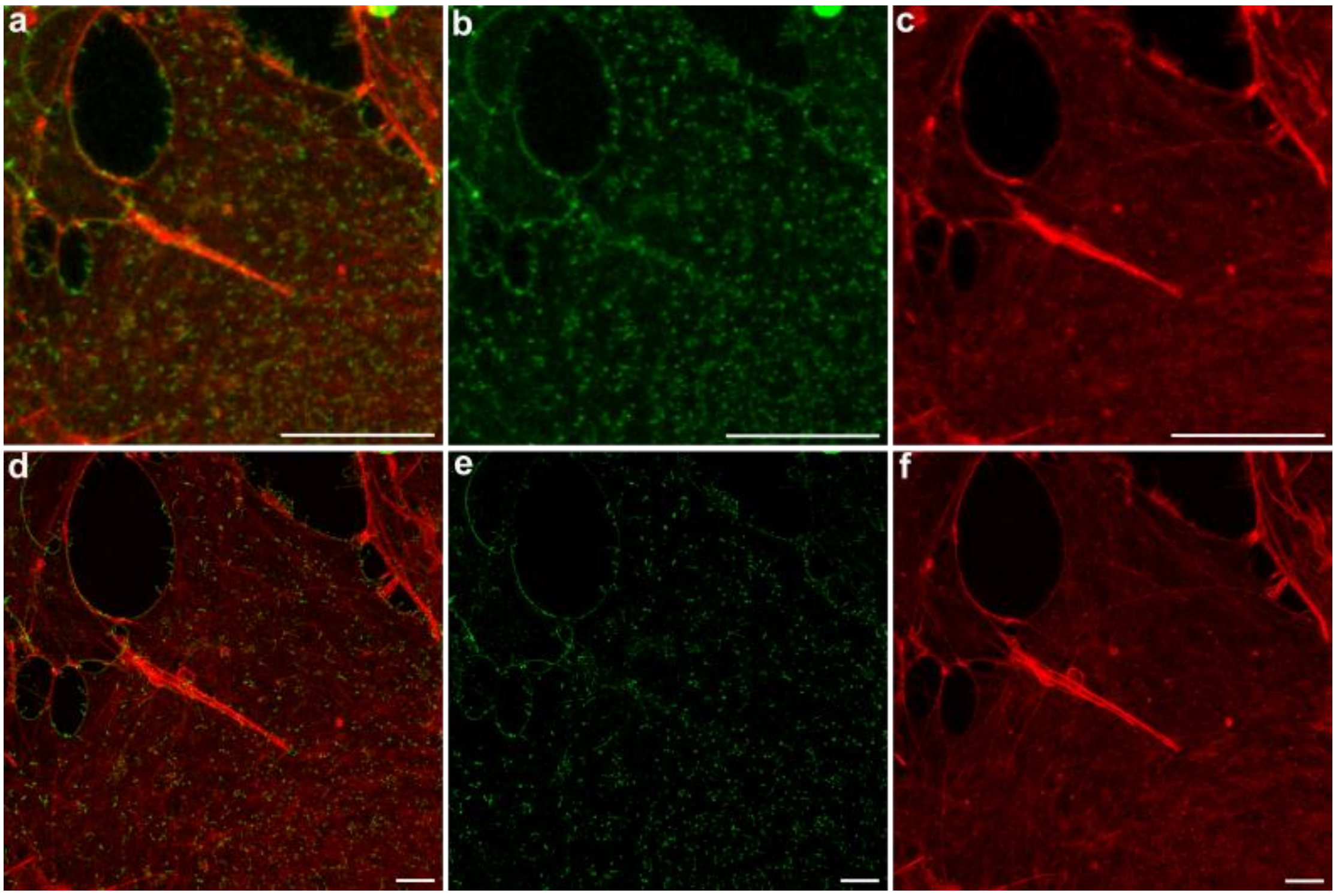

Figure S10. Multicolor images of cholesterol-rich domains and actin filaments stained with phalloidin in COS-7 cells using 4x ExM. a, Pre-expansion confocal fluorescence image of cholesterol-rich domains visualized with EGFP-D4 (green) or actin filaments ('Actin ExM', 561, red). **b**,**c**, The green and red channel in **a**. **d**, Post-expansion confocal fluorescence image of cholesterol-rich domains and actin obtained in the same cell after 4x ExM. **e**,**f**, The green and red channel in **d**. Representative images were selected from n= 3 independent samples. Scale bars, 20 μm.

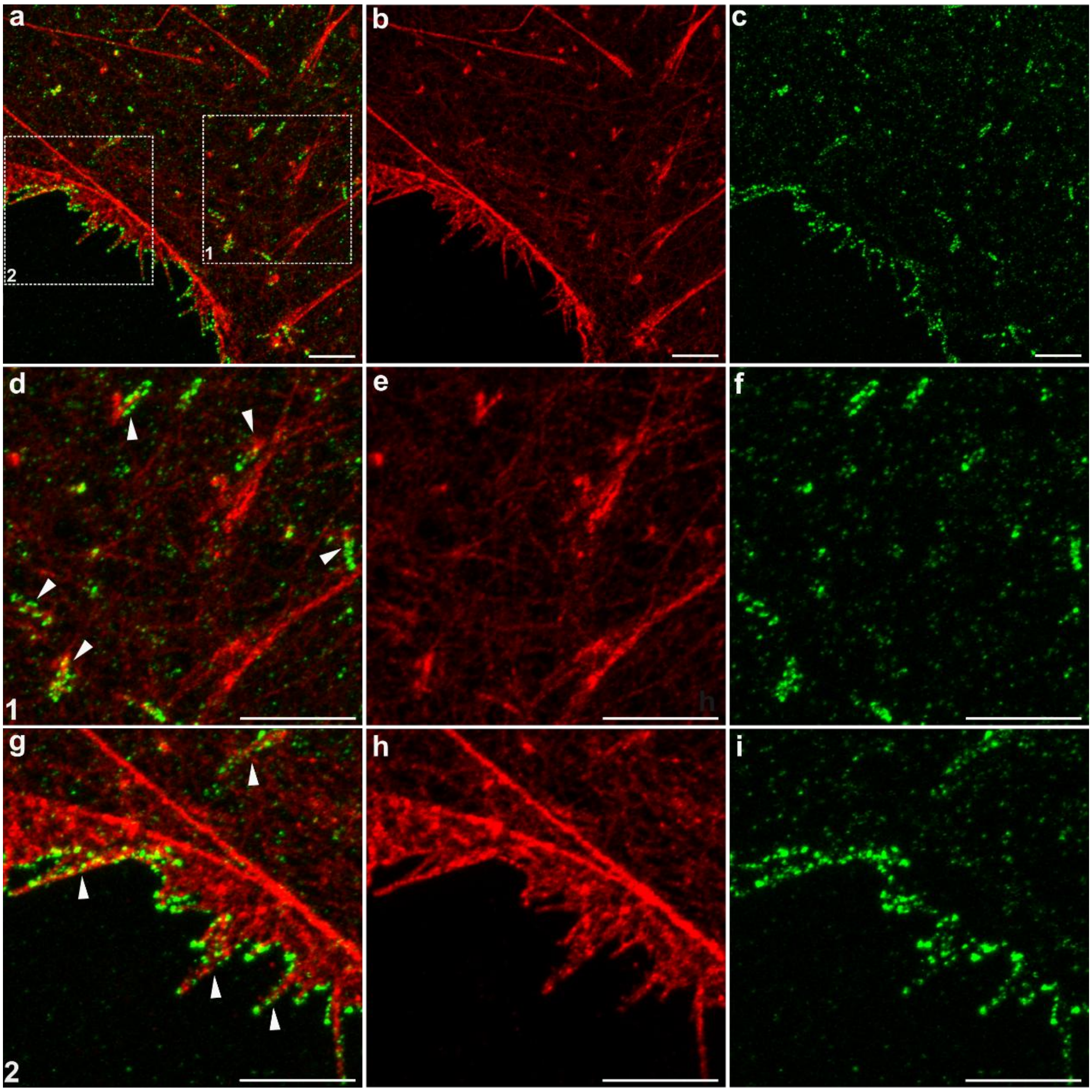

Figure S11. Nanoscale imaging of cholesterol-rich membrane domains costained with actin filaments using 4x ExM-Airyscan microscopy. a-c, Multi-color Airyscan SR images of cholesterol domains (EGFP, green) and actin ('Actin ExM', 561, red). **d-i**, Zoom-in views of the highlighted regions in panel **a**, showing microvilli. Representative images were from selected from n= 3 independent samples. Scale bars, 10 µm.

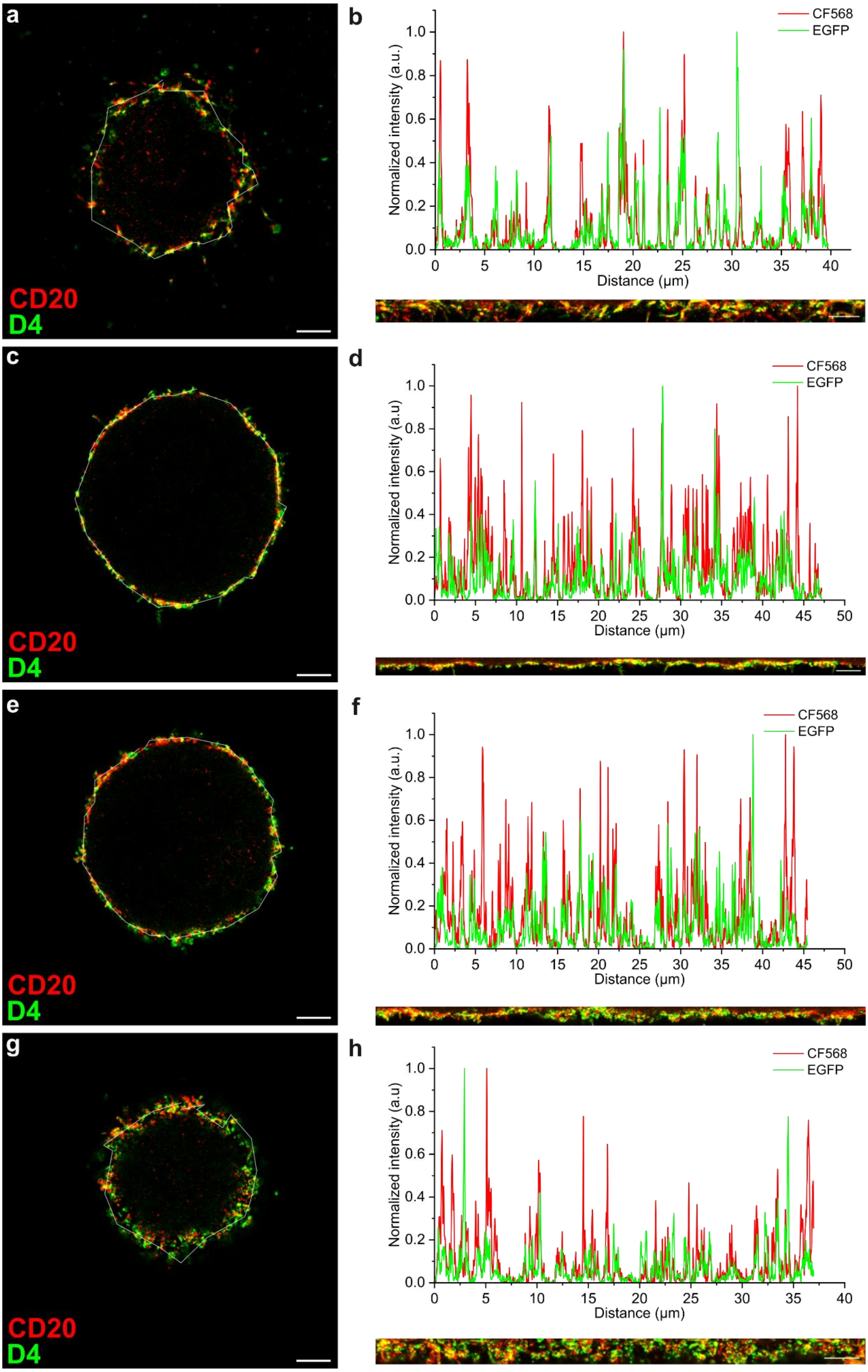

a
CD20
D4
b
CF568
EGFP
Normalized intensity (a.u.)
Distance (µm)
c
CD20
D4
d
CF568
EGFP
Normalized intensity (a.u)
Distance (µm)
e
CD20
D4
f
CF568
EGFP
Normalized intensity (a.u.)
Distance (µm)
g
CD20
D4
h
CF568
EGFP
Normalized intensity (a.u)
Distance (µm)

Figure S12. RTX and cholesterol-rich membrane domains colocalize on microvilli-like protrusions of Raji B cells. Representative z planes of the same cell shown in **figure 4a-e** are shown, highlighting the colocalization of RTX and cholesterol-rich membrane domains. **a**,**c,e,g**, Representative single z plane images of RTX-stained CD20 (CF568, red) and cholesterol-rich membrane domains (EGFP-D4, green). **b,d,f,h**, Corresponding normalized fluorescence intensity profiles along the white lines and straightened z-stack images of 10 z-slices along the circumference shown in **a,c,e,g** highlighting the colocalization (yellow) of CD20/RTX complexes and cholesterol-rich membrane domains on microvilli-like protrusions of B cells. Representative images were selected from n= 4 independent samples. Scale bars, 10 µm.

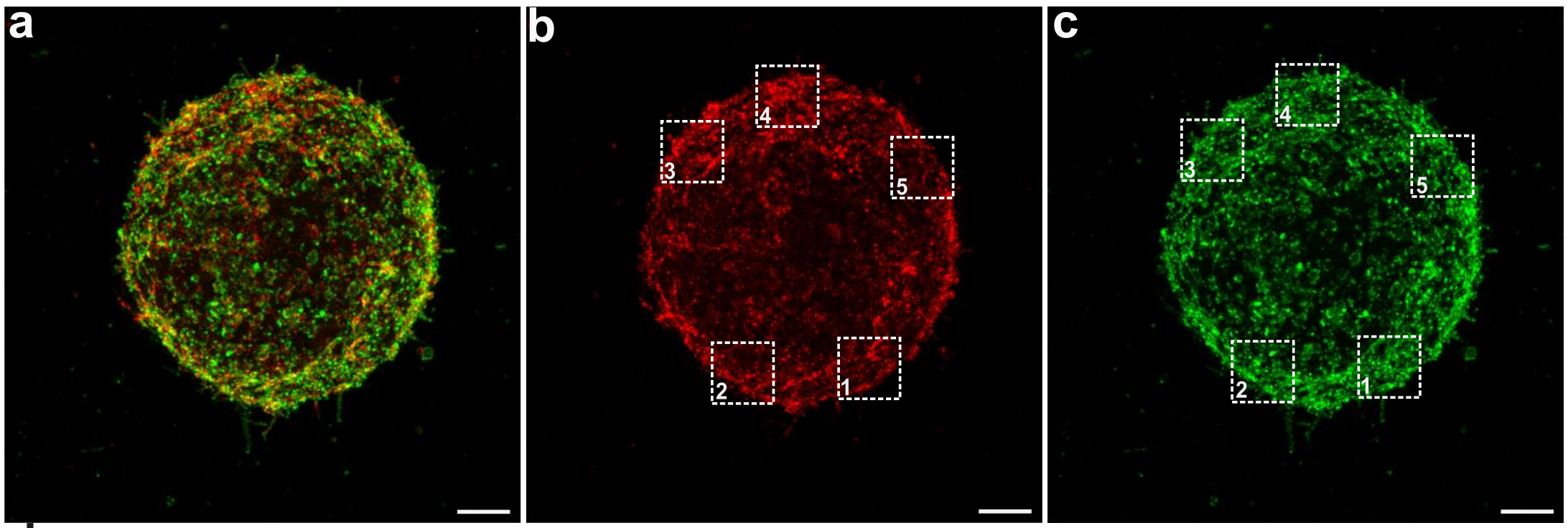

d

Area number in RTX-CF568 signal	Fluorescence intensity of indicated areas	Fluorescence intensity of the whole cell	Fluorescence ratio
1	45709.94	1432395.176	3.191%
2	48370.577	1432395.176	3.377%
3	64906.574	1432395.176	4.531%
4	67960.555	1432395.176	4.744%
5	38640.114	1432395.176	2.698%

e

Area number in EGFP-D4 signal	Fluorescence intensity of indicated areas	Fluorescence intensity of the whole cell	Fluorescence ratio
1	103031.693	2479123.232	4.156%
2	97228.092	2479123.232	3.922%
3	97858.973	2479123.232	3.947%
4	102056.905	2479123.232	4.117%
5	90306.499	2479123.232	3.643%

Figure S13. RTX labeling of CD20 induces polarization of Raji B cells, shown in figure 4a-e. B cell polarization was observed in RTX channel and not in EGFP-D4 channel. **a**-**c**, Airyscan super-resolution images of RTX-bound CD20 (red) and cholesterol-rich membrane domains (green) on a 4.1-fold expanded B cell. **d**, Quantification of fluorescence ratio of indicated regions shown in panel **b**, showing cell polarization. **e**, Quantification of the fluorescence ratio of indicated regions shown in panel **c**. Representative images were selected from n= 4 independent samples. Scale bars, 10 μm (**a**-**c**).

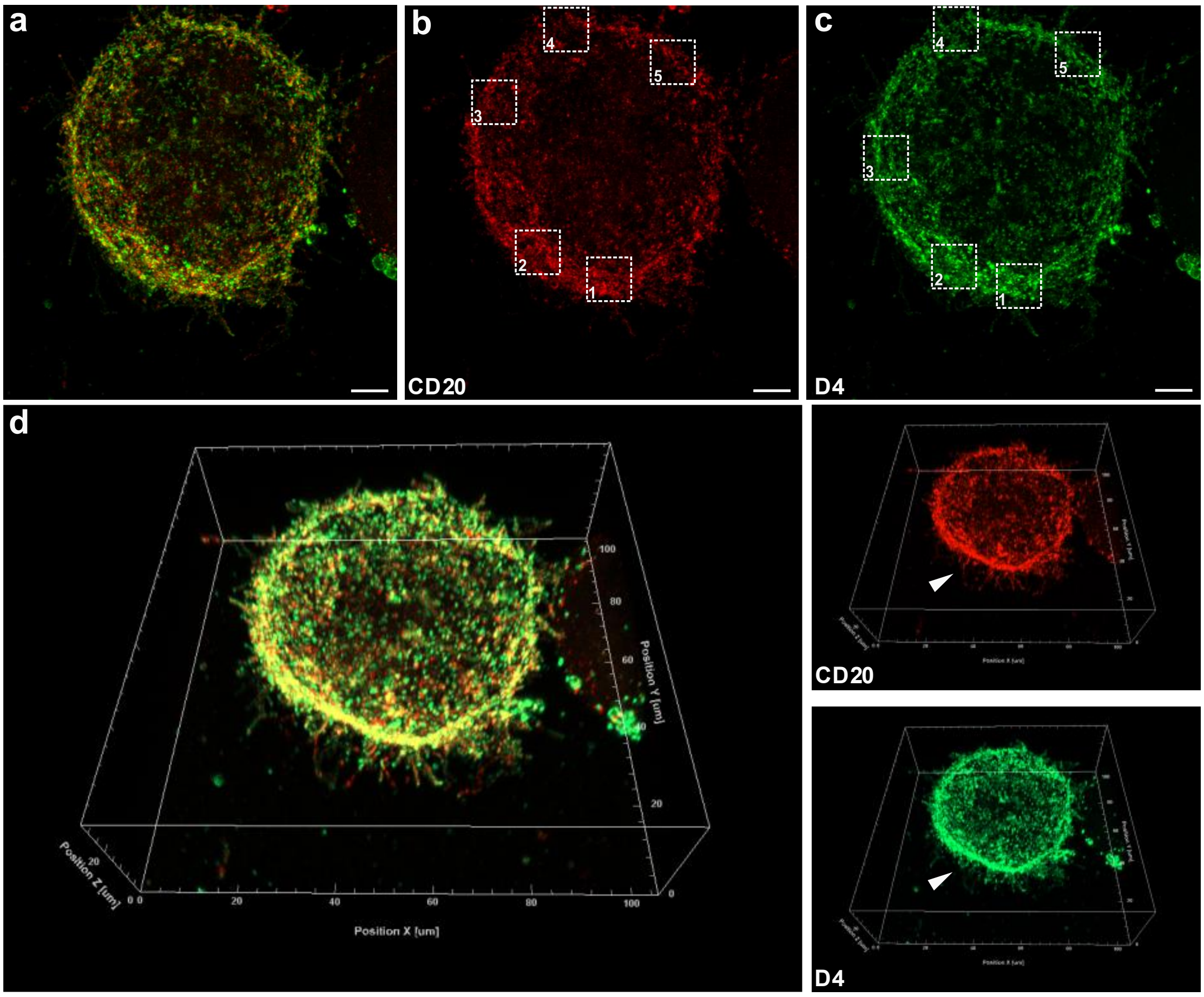

e

Area number in RTX-CF568 signal	Fluorescence intensity of indicated areas	Fluorescence intensity of the whole cell	Fluorescence ratio
1	61111.602	1427580.437	4.281%
2	64118.633	1427580.437	4.491%
3	42678.229	1427580.437	2.990%
4	32978.896	1427580.437	2.310%
5	33342.315	1427580.437	2.336%

f

Area number in EGFP-D4 signal	Fluorescence intensity of indicated areas	Fluorescence intensity of the whole cell	Fluorescence ratio
1	188976.91	3870963.297	4.882%
2	200991.706	3870963.297	5.192%
3	142073.782	3870963.297	3.670%
4	101980.904	3870963.297	2.635%
5	98206.097	3870963.297	2.537%

Figure S14. RTX labeling of CD20 induces polarization of Raji B cells. a-c, Airyscan super-resolution images of RTX-bound CD20 and cholesterol-rich membrane domains on a 4.1-fold expanded B cell. **d**, Corresponding 3D image of RTX-bound CD20 and cholesterol-rich membrane domains. B cell polarization is indicated by white arrows. **e**, Quantification of fluorescence ratio of indicated regions shown in panel **b**. **f**, Quantification of the fluorescence ratio of indicated regions shown in panel **c**. Representative images were selected from n= 4 independent samples. Scale bars, 10 μm (**a**-**c**).

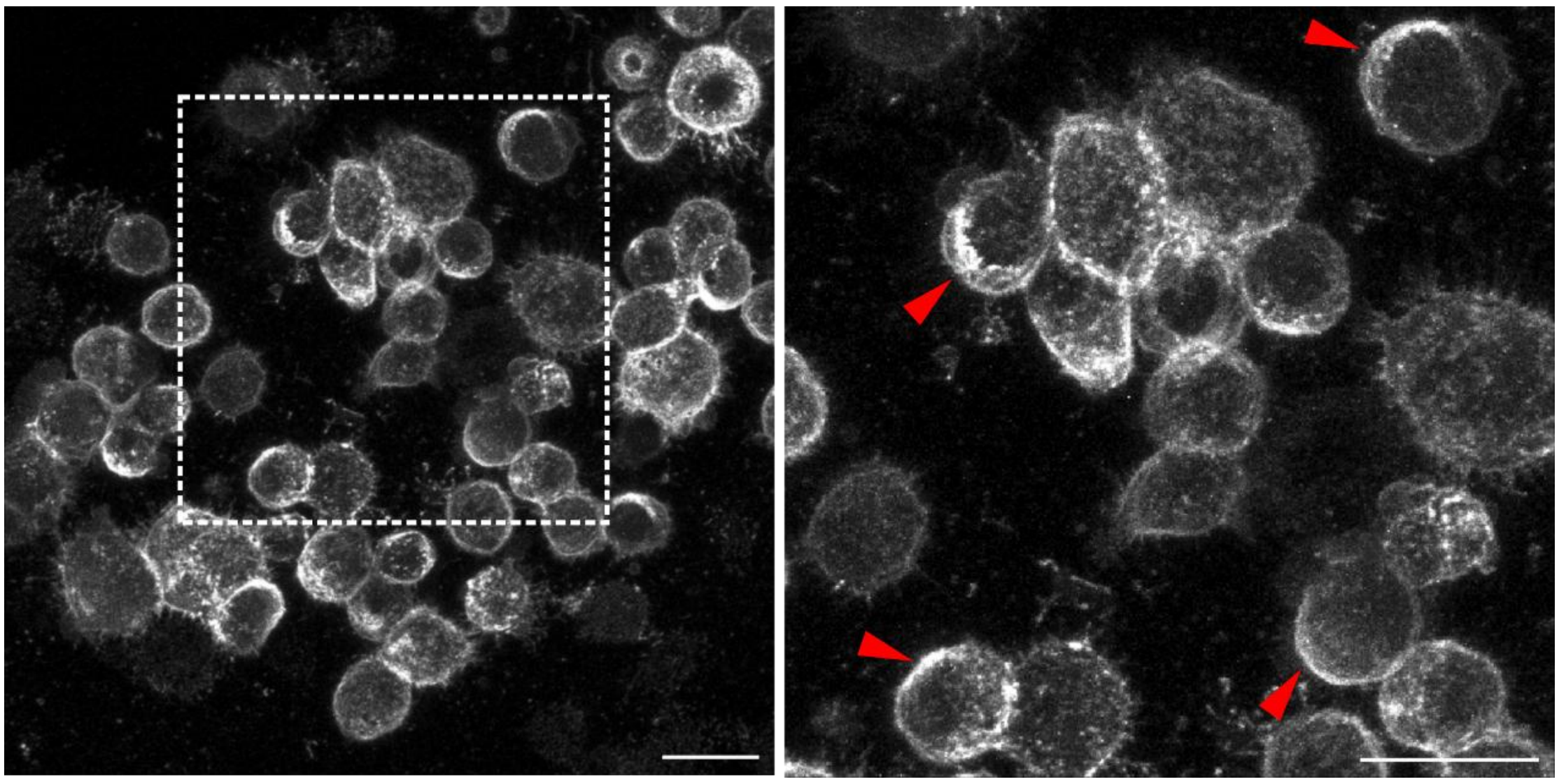

Figure S15. Confocal images of CD20 visualized by RTX-568 in Raji cells. Living Raji B cells were only stained with RTX-568, fixed and permeabilized. **a**,**b**, Confocal images of CD20-RTX using a 40x water objective (**a**) and zoom-in view of the highlighted region (**b**). Representative images were selected from n= 2 independent samples. Scale bars, 20 μm.

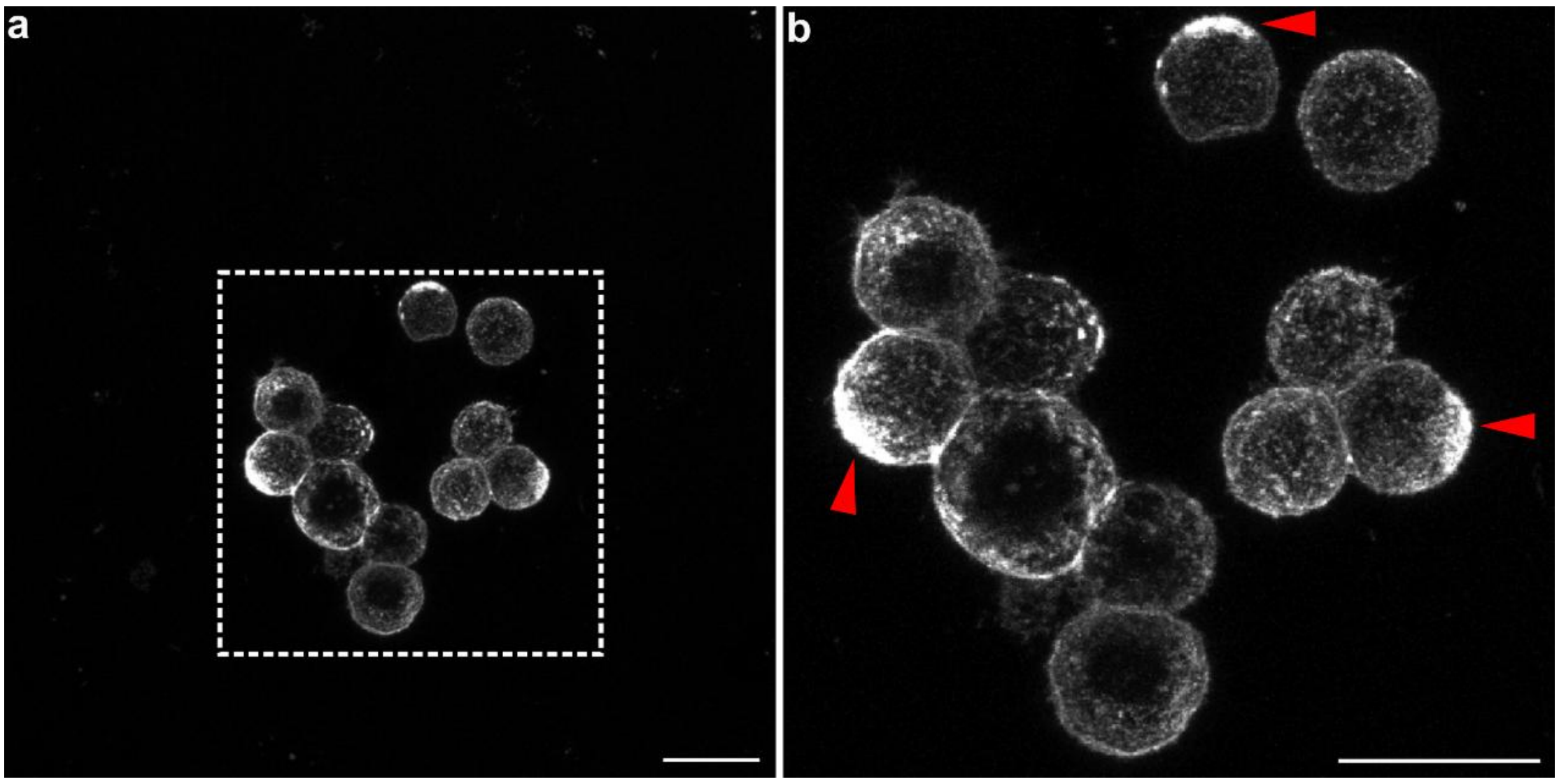

Figure S16. Confocal images of cholesterol-rich domains visualized by EGFP-D4 probes in Raji cells. Living Raji B cells were only stained with EGFP-D4, fixed and permeabilized. **a**,**b**, Confocal images of cholesterol-rich membrane domains using a 40x water objective (**a**) and zoom-in view of the highlighted region (**b**). Representative images were selected from n= 3 independent samples. Scale bars, 20 μm.

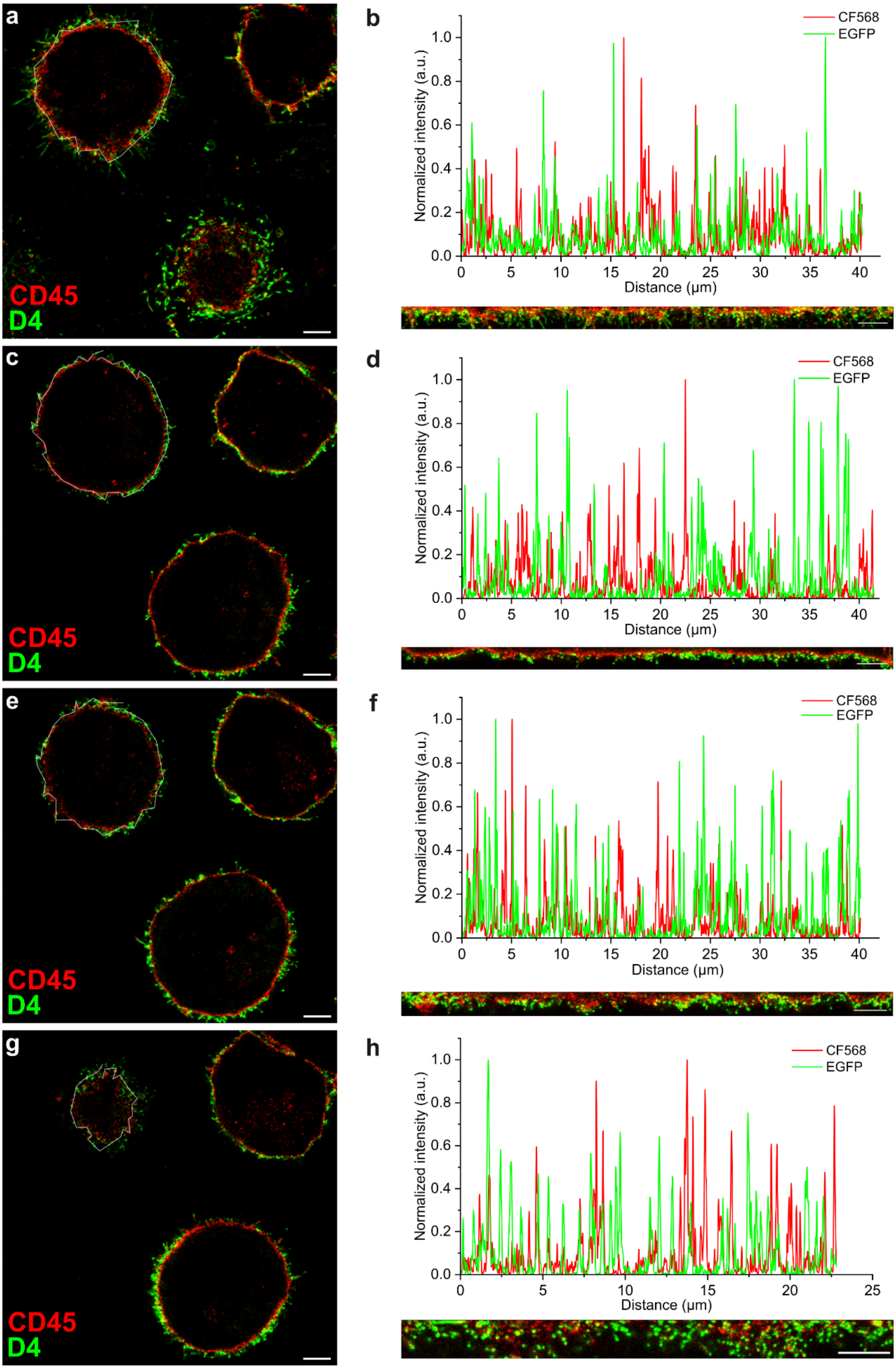
a
CD45
D4
b
CF568
EGFP
Normalized intensity (a.u.)
Distance (µm)
c
CD45
D4
d
CF568
EGFP
Normalized intensity (a.u.)
Distance (µm)
e
CD45
D4
f
CF568
EGFP
Normalized intensity (a.u.)
Distance (µm)
g
CD45
D4
h
CF568
EGFP
Normalized intensity (a.u.)
Distance (µm)

Figure S17. Anti-CD45 antibodies do not colocalize with cholesterol-rich membrane domains on microvilli-like protrusions of Raji B cells. **a**,**c**,**e**,**g**, Representative single z plane images of CD45 labeled with anti-CD45 antibodies (CF568. Red) and cholesterol-rich membrane domains (EGFP-D4, green). **b,d,f,h**, Corresponding normalized fluorescence intensity profiles along the white lines and straightened z-stack images of 10 z-slices along the circumference shown in **a,c,e,g** highlighting that anti-CD45 antibodies (red) do not colocalize with cholesterol-rich membrane domains on microvilli-like protrusions of B cells. Representative images were selected from n= 3 independent samples. Scale bars, 10 μm.

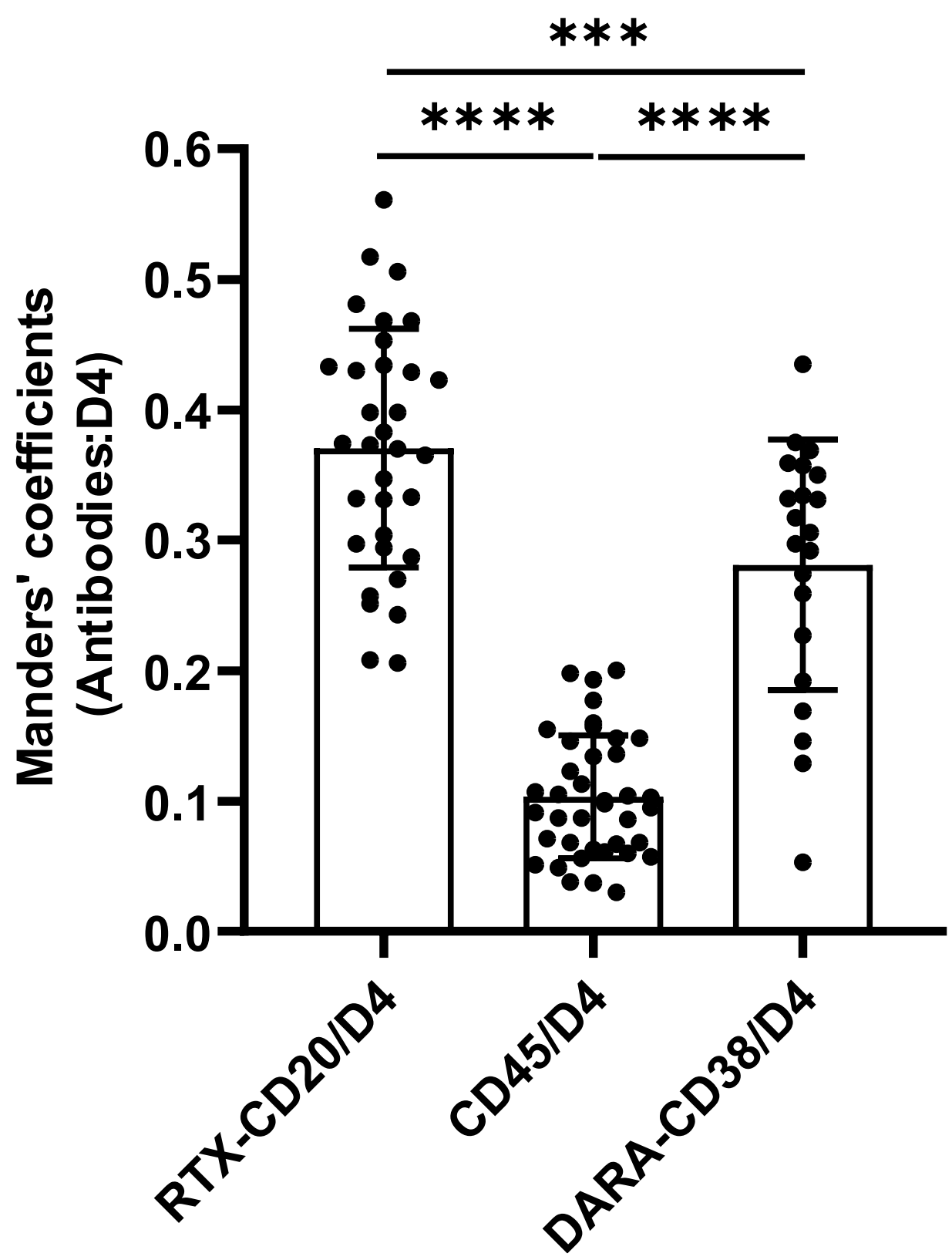

Figure S18. Comparison of colocalization coefficients of receptors and cholesterol-rich membrane domains. Bars represent the mean value and error bars represent the standard deviation. Statistical significance is assessed by one-way ANOVA test. **** $p<0.0001$, *** $p<0.001$. From left to right, mean values are 0.370 ± 0.092 (mean ± standard deviation), 0.103 ± 0.047, 0.281 ± 0.096, respectively.
