# Supplementary material for: Expansion Microscopy Reveals the Spatial Association of Therapeutic Antibody–Receptor Complexes With Cholesterol‐Rich Membrane Domains
Source: Angew Chem Int Ed Engl. 2026 Aug 24;65(42):e1206161. doi: 10.1002/anie.1206161 (PMC13653619; doi:10.1002/anie.1206161)
Supplement: Supplementary file 3 [file ANIE-65-e1206161-s003.zip › Raw dara of Manders analysis of CD20 and CD38 and CD45/CD45 and EGFP-raw data in total.docx]

**CD45 and EGFP-D4**

**20250910-S2**

**************************************************

Image A: C1-q1-1-SR__AiryscanProcessed-1.czi

Image B: C2-q1-1-SR__AiryscanProcessed-1.czi

Pearson's Coefficient:

r=0.42

Manders' Coefficients (original):

M1=0.914 (fraction of A overlapping B)

M2=0.959 (fraction of B overlapping A)

Manders' Coefficients (using threshold value of 132 for imgA and 1630 for imgB):

M1=0.091 (fraction of A overlapping B)

M2=0.129 (fraction of B overlapping A)

**************************************************

Image A: C1-q1-1-SR__AiryscanProcessed-1.czi

Image B: C2-q1-1-SR__AiryscanProcessed-1.czi

Pearson's Coefficient:

r=0.392

Manders' Coefficients (original):

M1=0.903 (fraction of A overlapping B)

M2=0.944 (fraction of B overlapping A)

Manders' Coefficients (using threshold value of 116 for imgA and 1077 for imgB):

M1=0.148 (fraction of A overlapping B)

M2=0.142 (fraction of B overlapping A)

**************************************************

Image A: C1-q1-2-SR__AiryscanProcessed-1.czi

Image B: C2-q1-2-SR__AiryscanProcessed-1.czi

Pearson's Coefficient:

r=0.413

Manders' Coefficients (original):

M1=0.895 (fraction of A overlapping B)

M2=0.926 (fraction of B overlapping A)

Manders' Coefficients (using threshold value of 240 for imgA and 1038 for imgB):

M1=0.107 (fraction of A overlapping B)

M2=0.163 (fraction of B overlapping A)

**************************************************

Image A: C1-q1-2-SR__AiryscanProcessed-1.czi

Image B: C2-q1-2-SR__AiryscanProcessed-1.czi

Pearson's Coefficient:

r=0.53

Manders' Coefficients (original):

M1=0.957 (fraction of A overlapping B)

M2=0.968 (fraction of B overlapping A)

Manders' Coefficients (using threshold value of 353 for imgA and 1979 for imgB):

M1=0.104 (fraction of A overlapping B)

M2=0.123 (fraction of B overlapping A)

**************************************************

Image A: C1-q1-2-SR__AiryscanProcessed-1.czi

Image B: C2-q1-2-SR__AiryscanProcessed-1.czi

Pearson's Coefficient:

r=0.353

Manders' Coefficients (original):

M1=0.81 (fraction of A overlapping B)

M2=0.911 (fraction of B overlapping A)

Manders' Coefficients (using threshold value of 192 for imgA and 785 for imgB):

M1=0.103 (fraction of A overlapping B)

M2=0.185 (fraction of B overlapping A)

**************************************************

Image A: C1-q2-1-SR__AiryscanProcessed-1.czi

Image B: C2-q2-1-SR__AiryscanProcessed-1.czi

Pearson's Coefficient:

r=0.35

Manders' Coefficients (original):

M1=0.847 (fraction of A overlapping B)

M2=0.945 (fraction of B overlapping A)

Manders' Coefficients (using threshold value of 224 for imgA and 1195 for imgB):

M1=0.057 (fraction of A overlapping B)

M2=0.07 (fraction of B overlapping A)

**************************************************

Image A: C1-q2-1-SR__AiryscanProcessed-1.czi

Image B: C2-q2-1-SR__AiryscanProcessed-1.czi

Pearson's Coefficient:

r=0.35

Manders' Coefficients (original):

M1=0.909 (fraction of A overlapping B)

M2=0.927 (fraction of B overlapping A)

Manders' Coefficients (using threshold value of 217 for imgA and 738 for imgB):

M1=0.134 (fraction of A overlapping B)

M2=0.062 (fraction of B overlapping A)

**************************************************

**************************************************

Image A: C1-q2-1-SR__AiryscanProcessed-1.czi

Image B: C2-q2-1-SR__AiryscanProcessed-1.czi

Pearson's Coefficient:

r=0.269

Manders' Coefficients (original):

M1=0.827 (fraction of A overlapping B)

M2=0.873 (fraction of B overlapping A)

Manders' Coefficients (using threshold value of 129 for imgA and 623 for imgB):

M1=0.049 (fraction of A overlapping B)

M2=0.087 (fraction of B overlapping A)

**************************************************

Image A: C1-q2-2-SR__AiryscanProcessed-1.czi

Image B: C2-q2-2-SR__AiryscanProcessed-1.czi

Pearson's Coefficient:

r=0.315

Manders' Coefficients (original):

M1=0.811 (fraction of A overlapping B)

M2=0.924 (fraction of B overlapping A)

Manders' Coefficients (using threshold value of 134 for imgA and 584 for imgB):

M1=0.095 (fraction of A overlapping B)

M2=0.134 (fraction of B overlapping A)

**************************************************

Image A: C1-q2-2-SR__AiryscanProcessed-1.czi

Image B: C2-q2-2-SR__AiryscanProcessed-1.czi

Pearson's Coefficient:

r=0.371

Manders' Coefficients (original):

M1=0.851 (fraction of A overlapping B)

M2=0.92 (fraction of B overlapping A)

Manders' Coefficients (using threshold value of 155 for imgA and 605 for imgB):

M1=0.155 (fraction of A overlapping B)

M2=0.134 (fraction of B overlapping A)

**************************************************

Image A: C1-q3-1-SR__AiryscanProcessed-1.czi

Image B: C2-q3-1-SR__AiryscanProcessed-1.czi

Pearson's Coefficient:

r=0.374

Manders' Coefficients (original):

M1=0.81 (fraction of A overlapping B)

M2=0.869 (fraction of B overlapping A)

Manders' Coefficients (using threshold value of 177 for imgA and 382 for imgB):

M1=0.193 (fraction of A overlapping B)

M2=0.101 (fraction of B overlapping A)

**************************************************

Image A: C1-q3-1-SR__AiryscanProcessed-1.czi

Image B: C2-q3-1-SR__AiryscanProcessed-1.czi

Pearson's Coefficient:

r=0.285

Manders' Coefficients (original):

M1=0.787 (fraction of A overlapping B)

M2=0.916 (fraction of B overlapping A)

Manders' Coefficients (using threshold value of 176 for imgA and 735 for imgB):

M1=0.068 (fraction of A overlapping B)

M2=0.085 (fraction of B overlapping A)

**************************************************

Image A: C1-q3-2-SR__AiryscanProcessed.czi

Image B: C2-q3-2-SR__AiryscanProcessed.czi

Pearson's Coefficient:

r=0.419

Manders' Coefficients (original):

M1=0.912 (fraction of A overlapping B)

M2=0.939 (fraction of B overlapping A)

Manders' Coefficients (using threshold value of 248 for imgA and 1259 for imgB):

M1=0.087 (fraction of A overlapping B)

M2=0.098 (fraction of B overlapping A)

**************************************************

Image A: C1-q4-1-SR__AiryscanProcessed-1.czi

Image B: C2-q4-1-SR__AiryscanProcessed-1.czi

Pearson's Coefficient:

r=0.406

Manders' Coefficients (original):

M1=0.946 (fraction of A overlapping B)

M2=0.954 (fraction of B overlapping A)

Manders' Coefficients (using threshold value of 228 for imgA and 1094 for imgB):

M1=0.198 (fraction of A overlapping B)

M2=0.14 (fraction of B overlapping A)

**************************************************

Image A: C1-q4-1-SR__AiryscanProcessed-1.czi

Image B: C2-q4-1-SR__AiryscanProcessed-1.czi

Pearson's Coefficient:

r=0.376

Manders' Coefficients (original):

M1=0.925 (fraction of A overlapping B)

M2=0.955 (fraction of B overlapping A)

Manders' Coefficients (using threshold value of 275 for imgA and 1112 for imgB):

M1=0.087 (fraction of A overlapping B)

M2=0.076 (fraction of B overlapping A)

**************************************************

Image A: C1-q4-1-SR__AiryscanProcessed-1.czi

Image B: C2-q4-1-SR__AiryscanProcessed-1.czi

Pearson's Coefficient:

r=0.391

Manders' Coefficients (original):

M1=0.927 (fraction of A overlapping B)

M2=0.941 (fraction of B overlapping A)

Manders' Coefficients (using threshold value of 231 for imgA and 967 for imgB):

M1=0.086 (fraction of A overlapping B)

M2=0.118 (fraction of B overlapping A)

**************************************************

Image A: C1-q4-1-SR__AiryscanProcessed-1.czi

Image B: C2-q4-1-SR__AiryscanProcessed-1.czi

Pearson's Coefficient:

r=0.338

Manders' Coefficients (original):

M1=0.91 (fraction of A overlapping B)

M2=0.932 (fraction of B overlapping A)

Manders' Coefficients (using threshold value of 214 for imgA and 877 for imgB):

M1=0.068 (fraction of A overlapping B)

M2=0.06 (fraction of B overlapping A)

**************************************************

Image A: C1-q4-2-SR__AiryscanProcessed-1.czi

Image B: C2-q4-2-SR__AiryscanProcessed-1.czi

Pearson's Coefficient:

r=0.479

Manders' Coefficients (original):

M1=0.92 (fraction of A overlapping B)

M2=0.929 (fraction of B overlapping A)

Manders' Coefficients (using threshold value of 208 for imgA and 1292 for imgB):

M1=0.063 (fraction of A overlapping B)

M2=0.16 (fraction of B overlapping A)

**************************************************

Image A: C1-q4-2-SR__AiryscanProcessed-1.czi

Image B: C2-q4-2-SR__AiryscanProcessed-1.czi

Pearson's Coefficient:

r=0.479

Manders' Coefficients (original):

M1=0.92 (fraction of A overlapping B)

M2=0.929 (fraction of B overlapping A)

Manders' Coefficients (using threshold value of 208 for imgA and 1033 for imgB):

M1=0.105 (fraction of A overlapping B)

M2=0.155 (fraction of B overlapping A)

**************************************************

Image A: C1-q5-1-SR__AiryscanProcessed-1.czi

Image B: C2-q5-1-SR__AiryscanProcessed-1.czi

Pearson's Coefficient:

r=0.5

Manders' Coefficients (original):

M1=0.917 (fraction of A overlapping B)

M2=0.936 (fraction of B overlapping A)

Manders' Coefficients (using threshold value of 302 for imgA and 1477 for imgB):

M1=0.146 (fraction of A overlapping B)

M2=0.064 (fraction of B overlapping A)

**************************************************

Image A: C1-q5-1-SR__AiryscanProcessed-1.czi

Image B: C2-q5-1-SR__AiryscanProcessed-1.czi

Pearson's Coefficient:

r=0.485

Manders' Coefficients (original):

M1=0.947 (fraction of A overlapping B)

M2=0.936 (fraction of B overlapping A)

Manders' Coefficients (using threshold value of 177 for imgA and 1196 for imgB):

M1=0.2 (fraction of A overlapping B)

M2=0.182 (fraction of B overlapping A)

**************************************************

Image A: C1-q5-2-SR__AiryscanProcessed.czi

Image B: C2-q5-2-SR__AiryscanProcessed.czi

Pearson's Coefficient:

r=0.401

Manders' Coefficients (original):

M1=0.896 (fraction of A overlapping B)

M2=0.905 (fraction of B overlapping A)

Manders' Coefficients (using threshold value of 193 for imgA and 1621 for imgB):

M1=0.038 (fraction of A overlapping B)

M2=0.055 (fraction of B overlapping A)

**20250910-S3**

**************************************************

Image A: C1-q1-1-SR__AiryscanProcessed-1.czi

Image B: C2-q1-1-SR__AiryscanProcessed-1.czi

Pearson's Coefficient:

r=0.404

Manders' Coefficients (original):

M1=0.918 (fraction of A overlapping B)

M2=0.958 (fraction of B overlapping A)

Manders' Coefficients (using threshold value of 335 for imgA and 1023 for imgB):

M1=0.123 (fraction of A overlapping B)

M2=0.081 (fraction of B overlapping A)

**************************************************

Image A: C1-q1-1-SR__AiryscanProcessed-1.czi

Image B: C2-q1-1-SR__AiryscanProcessed-1.czi

Pearson's Coefficient:

r=0.398

Manders' Coefficients (original):

M1=0.89 (fraction of A overlapping B)

M2=0.938 (fraction of B overlapping A)

Manders' Coefficients (using threshold value of 292 for imgA and 911 for imgB):

M1=0.1 (fraction of A overlapping B)

M2=0.069 (fraction of B overlapping A)

**************************************************

Image A: C1-q1-2-SR__AiryscanProcessed-1.czi

Image B: C2-q1-2-SR__AiryscanProcessed-1.czi

Pearson's Coefficient:

r=0.366

Manders' Coefficients (original):

M1=0.773 (fraction of A overlapping B)

M2=0.891 (fraction of B overlapping A)

Manders' Coefficients (using threshold value of 203 for imgA and 627 for imgB):

M1=0.16 (fraction of A overlapping B)

M2=0.12 (fraction of B overlapping A)

**************************************************

Image A: C1-q1-2-SR__AiryscanProcessed-1.czi

Image B: C2-q1-2-SR__AiryscanProcessed-1.czi

Pearson's Coefficient:

r=0.299

Manders' Coefficients (original):

M1=0.814 (fraction of A overlapping B)

M2=0.932 (fraction of B overlapping A)

Manders' Coefficients (using threshold value of 234 for imgA and 738 for imgB):

M1=0.056 (fraction of A overlapping B)

M2=0.13 (fraction of B overlapping A)

**************************************************

Image A: C1-q1-3-SR__AiryscanProcessed.czi

Image B: C2-q1-3-SR__AiryscanProcessed.czi

Pearson's Coefficient:

r=0.426

Manders' Coefficients (original):

M1=0.875 (fraction of A overlapping B)

M2=0.948 (fraction of B overlapping A)

Manders' Coefficients (using threshold value of 333 for imgA and 2615 for imgB):

M1=0.051 (fraction of A overlapping B)

M2=0.041 (fraction of B overlapping A)

**************************************************

Image A: C1-q1-4-SR__AiryscanProcessed.czi

Image B: C2-q1-4-SR__AiryscanProcessed.czi

Pearson's Coefficient:

r=0.345

Manders' Coefficients (original):

M1=0.874 (fraction of A overlapping B)

M2=0.964 (fraction of B overlapping A)

Manders' Coefficients (using threshold value of 465 for imgA and 1307 for imgB):

M1=0.071 (fraction of A overlapping B)

M2=0.056 (fraction of B overlapping A)

**************************************************

Image A: C1-q2-1-SR__AiryscanProcessed-1.czi

Image B: C2-q2-1-SR__AiryscanProcessed-1.czi

Pearson's Coefficient:

r=0.424

Manders' Coefficients (original):

M1=0.913 (fraction of A overlapping B)

M2=0.953 (fraction of B overlapping A)

Manders' Coefficients (using threshold value of 387 for imgA and 605 for imgB):

M1=0.157 (fraction of A overlapping B)

M2=0.127 (fraction of B overlapping A)

20250910-S8

**************************************************

Image A: C1-q4-1-SR__AiryscanProcessed-1.czi

Image B: C2-q4-1-SR__AiryscanProcessed-1.czi

Pearson's Coefficient:

r=0.487

Manders' Coefficients (original):

M1=0.97 (fraction of A overlapping B)

M2=0.986 (fraction of B overlapping A)

Manders' Coefficients (using threshold value of 1252 for imgA and 1612 for imgB):

M1=0.067 (fraction of A overlapping B)

M2=0.074 (fraction of B overlapping A)

**************************************************

Image A: C1-q4-1-SR__AiryscanProcessed-1.czi

Image B: C2-q4-1-SR__AiryscanProcessed-1.czi

Pearson's Coefficient:

r=0.44

Manders' Coefficients (original):

M1=0.966 (fraction of A overlapping B)

M2=0.979 (fraction of B overlapping A)

Manders' Coefficients (using threshold value of 1141 for imgA and 1436 for imgB):

M1=0.061 (fraction of A overlapping B)

M2=0.057 (fraction of B overlapping A)

**************************************************

Image A: C1-q4-1-SR__AiryscanProcessed-1.czi

Image B: C2-q4-1-SR__AiryscanProcessed-1.czi

Pearson's Coefficient:

r=0.403

Manders' Coefficients (original):

M1=0.958 (fraction of A overlapping B)

M2=0.985 (fraction of B overlapping A)

Manders' Coefficients (using threshold value of 1318 for imgA and 1028 for imgB):

M1=0.06 (fraction of A overlapping B)

M2=0.028 (fraction of B overlapping A)

**************************************************

Image A: C1-q4-2-SR__AiryscanProcessed-1.czi

Image B: C2-q4-2-SR__AiryscanProcessed-1.czi

Pearson's Coefficient:

r=0.441

Manders' Coefficients (original):

M1=0.964 (fraction of A overlapping B)

M2=0.983 (fraction of B overlapping A)

Manders' Coefficients (using threshold value of 1185 for imgA and 1055 for imgB):

M1=0.098 (fraction of A overlapping B)

M2=0.016 (fraction of B overlapping A)

**************************************************

Image A: C1-q4-2-SR__AiryscanProcessed-1.czi

Image B: C2-q4-2-SR__AiryscanProcessed-1.czi

Pearson's Coefficient:

r=0.448

Manders' Coefficients (original):

M1=0.953 (fraction of A overlapping B)

M2=0.971 (fraction of B overlapping A)

Manders' Coefficients (using threshold value of 1085 for imgA and 979 for imgB):

M1=0.136 (fraction of A overlapping B)

M2=0.03 (fraction of B overlapping A)

**************************************************

Image A: C1-q5-1-SR__AiryscanProcessed-1.czi

Image B: C2-q5-1-SR__AiryscanProcessed-1.czi

Pearson's Coefficient:

r=0.476

Manders' Coefficients (original):

M1=0.975 (fraction of A overlapping B)

M2=0.984 (fraction of B overlapping A)

Manders' Coefficients (using threshold value of 1621 for imgA and 2161 for imgB):

M1=0.03 (fraction of A overlapping B)

M2=0.013 (fraction of B overlapping A)

**************************************************

Image A: C1-q5-1-SR__AiryscanProcessed-1.czi

Image B: C2-q5-1-SR__AiryscanProcessed-1.czi

Pearson's Coefficient:

r=0.54

Manders' Coefficients (original):

M1=0.972 (fraction of A overlapping B)

M2=0.981 (fraction of B overlapping A)

Manders' Coefficients (using threshold value of 1153 for imgA and 1546 for imgB):

M1=0.113 (fraction of A overlapping B)

M2=0.064 (fraction of B overlapping A)

**************************************************

Image A: C1-q7-1-SR__AiryscanProcessed-1.czi

Image B: C2-q7-1-SR__AiryscanProcessed-1.czi

Pearson's Coefficient:

r=0.501

Manders' Coefficients (original):

M1=0.92 (fraction of A overlapping B)

M2=0.972 (fraction of B overlapping A)

Manders' Coefficients (using threshold value of 1189 for imgA and 910 for imgB):

M1=0.148 (fraction of A overlapping B)

M2=0.091 (fraction of B overlapping A)

**************************************************

Image A: C1-q7-1-SR__AiryscanProcessed-1.czi

Image B: C2-q7-1-SR__AiryscanProcessed-1.czi

Pearson's Coefficient:

r=0.439

Manders' Coefficients (original):

M1=0.92 (fraction of A overlapping B)

M2=0.98 (fraction of B overlapping A)

Manders' Coefficients (using threshold value of 1428 for imgA and 1090 for imgB):

M1=0.177 (fraction of A overlapping B)

M2=0.108 (fraction of B overlapping A)

**************************************************

Image A: C1-q7-2-SR__AiryscanProcessed.czi

Image B: C2-q7-2-SR__AiryscanProcessed.czi

Pearson's Coefficient:

r=0.572

Manders' Coefficients (original):

M1=0.931 (fraction of A overlapping B)

M2=0.982 (fraction of B overlapping A)

Manders' Coefficients (using threshold value of 1499 for imgA and 3326 for imgB):

M1=0.037 (fraction of A overlapping B)

M2=0.035 (fraction of B overlapping A)
