# Supplementary material for: Expansion Microscopy Reveals the Spatial Association of Therapeutic Antibody–Receptor Complexes With Cholesterol‐Rich Membrane Domains
Source: Angew Chem Int Ed Engl. 2026 Aug 24;65(42):e1206161. doi: 10.1002/anie.1206161 (PMC13653619; doi:10.1002/anie.1206161)
Supplement: Supplementary file 3 [file ANIE-65-e1206161-s003.zip › Raw dara of Manders analysis of CD20 and CD38 and CD45/DARA-D4-raw data in total.docx]

**DARA-CD38 and EGFP-D4**

**20250814-S3**

**************************************************

Image A: C1-q1-2-SR__AiryscanProcessed.czi

Image B: C2-q1-2-SR__AiryscanProcessed.czi

Pearson's Coefficient:

r=0.622

Manders' Coefficients (original):

M1=0.957 (fraction of A overlapping B)

M2=0.983 (fraction of B overlapping A)

Manders' Coefficients (using threshold value of 748 for imgA and 768 for imgB):

M1=0.331 (fraction of A overlapping B)

M2=0.256 (fraction of B overlapping A)

**************************************************

Image A: C1-q1-3-SR__AiryscanProcessed.czi

Image B: C2-q1-3-SR__AiryscanProcessed.czi

Pearson's Coefficient:

r=0.616

Manders' Coefficients (original):

M1=0.963 (fraction of A overlapping B)

M2=0.98 (fraction of B overlapping A)

Manders' Coefficients (using threshold value of 765 for imgA and 1361 for imgB):

M1=0.192 (fraction of A overlapping B)

M2=0.132 (fraction of B overlapping A)

**************************************************

Image A: C1-q2-1-SR__AiryscanProcessed.czi

Image B: C2-q2-1-SR__AiryscanProcessed.czi

Pearson's Coefficient:

r=0.606

Manders' Coefficients (original):

M1=0.966 (fraction of A overlapping B)

M2=0.984 (fraction of B overlapping A)

Manders' Coefficients (using threshold value of 805 for imgA and 1221 for imgB):

M1=0.146 (fraction of A overlapping B)

M2=0.31 (fraction of B overlapping A)

**************************************************

Image A: C1-q4-1-SR__AiryscanProcessed-1.czi

Image B: C2-q4-1-SR__AiryscanProcessed-1.czi

Pearson's Coefficient:

r=0.568

Manders' Coefficients (original):

M1=0.955 (fraction of A overlapping B)

M2=0.97 (fraction of B overlapping A)

Manders' Coefficients (using threshold value of 372 for imgA and 944 for imgB):

M1=0.317 (fraction of A overlapping B)

M2=0.334 (fraction of B overlapping A)

**************************************************

Image A: C1-q4-1-SR__AiryscanProcessed-1.czi

Image B: C2-q4-1-SR__AiryscanProcessed-1.czi

Pearson's Coefficient:

r=0.588

Manders' Coefficients (original):

M1=0.967 (fraction of A overlapping B)

M2=0.987 (fraction of B overlapping A)

Manders' Coefficients (using threshold value of 976 for imgA and 1272 for imgB):

M1=0.129 (fraction of A overlapping B)

M2=0.227 (fraction of B overlapping A)

**************************************************

Image A: C1-q5-1-SR__AiryscanProcessed.czi

Image B: C2-q5-1-SR__AiryscanProcessed.czi

Pearson's Coefficient:

r=0.697

Manders' Coefficients (original):

M1=0.967 (fraction of A overlapping B)

M2=0.982 (fraction of B overlapping A)

Manders' Coefficients (using threshold value of 610 for imgA and 1210 for imgB):

M1=0.357 (fraction of A overlapping B)

M2=0.493 (fraction of B overlapping A)

**************************************************

Image A: C1-q5-2-SR__AiryscanProcessed.czi

Image B: C2-q5-2-SR__AiryscanProcessed.czi

Pearson's Coefficient:

r=0.655

Manders' Coefficients (original):

M1=0.969 (fraction of A overlapping B)

M2=0.985 (fraction of B overlapping A)

Manders' Coefficients (using threshold value of 874 for imgA and 1039 for imgB):

M1=0.35 (fraction of A overlapping B)

M2=0.277 (fraction of B overlapping A)

**20250814-S4**

**************************************************

Image A: C1-q1-1-SR__AiryscanProcessed.czi

Image B: C2-q1-1-SR__AiryscanProcessed.czi

Pearson's Coefficient:

r=0.627

Manders' Coefficients (original):

M1=0.963 (fraction of A overlapping B)

M2=0.97 (fraction of B overlapping A)

Manders' Coefficients (using threshold value of 294 for imgA and 1966 for imgB):

M1=0.053 (fraction of A overlapping B)

M2=0.224 (fraction of B overlapping A)

**************************************************

Image A: C1-q1-2-SR__AiryscanProcessed-1.czi

Image B: C2-q1-2-SR__AiryscanProcessed-1.czi

Pearson's Coefficient:

r=0.683

Manders' Coefficients (original):

M1=0.975 (fraction of A overlapping B)

M2=0.966 (fraction of B overlapping A)

Manders' Coefficients (using threshold value of 284 for imgA and 1397 for imgB):

M1=0.369 (fraction of A overlapping B)

M2=0.187 (fraction of B overlapping A)

**************************************************

Image A: C1-q1-2-SR__AiryscanProcessed.czi

Image B: C2-q1-2-SR__AiryscanProcessed.czi

Pearson's Coefficient:

r=0.693

Manders' Coefficients (original):

M1=0.966 (fraction of A overlapping B)

M2=0.962 (fraction of B overlapping A)

Manders' Coefficients (using threshold value of 278 for imgA and 1586 for imgB):

M1=0.292 (fraction of A overlapping B)

M2=0.195 (fraction of B overlapping A)

**************************************************

Image A: C1-q1-3-SR__AiryscanProcessed.czi

Image B: C2-q1-3-SR__AiryscanProcessed.czi

Pearson's Coefficient:

r=0.651

Manders' Coefficients (original):

M1=0.939 (fraction of A overlapping B)

M2=0.958 (fraction of B overlapping A)

Manders' Coefficients (using threshold value of 259 for imgA and 1220 for imgB):

M1=0.375 (fraction of A overlapping B)

M2=0.25 (fraction of B overlapping A)

**************************************************

Image A: C1-q2-1-SR__AiryscanProcessed.czi

Image B: C2-q2-1-SR__AiryscanProcessed.czi

Pearson's Coefficient:

r=0.661

Manders' Coefficients (original):

M1=0.952 (fraction of A overlapping B)

M2=0.979 (fraction of B overlapping A)

Manders' Coefficients (using threshold value of 694 for imgA and 948 for imgB):

M1=0.435 (fraction of A overlapping B)

M2=0.199 (fraction of B overlapping A)

**************************************************

Image A: C1-q2-2-SR__AiryscanProcessed.czi

Image B: C2-q2-2-SR__AiryscanProcessed.czi

Pearson's Coefficient:

r=0.704

Manders' Coefficients (original):

M1=0.969 (fraction of A overlapping B)

M2=0.985 (fraction of B overlapping A)

Manders' Coefficients (using threshold value of 782 for imgA and 1616 for imgB):

M1=0.332 (fraction of A overlapping B)

M2=0.395 (fraction of B overlapping A)

**************************************************

Image A: C1-q3-1-SR__AiryscanProcessed.czi

Image B: C2-q3-1-SR__AiryscanProcessed.czi

Pearson's Coefficient:

r=0.528

Manders' Coefficients (original):

M1=0.951 (fraction of A overlapping B)

M2=0.977 (fraction of B overlapping A)

Manders' Coefficients (using threshold value of 548 for imgA and 988 for imgB):

M1=0.169 (fraction of A overlapping B)

M2=0.129 (fraction of B overlapping A)

**************************************************

Image A: C1-q4-2-SR__AiryscanProcessed-1.czi

Image B: C2-q4-2-SR__AiryscanProcessed-1.czi

Pearson's Coefficient:

r=0.645

Manders' Coefficients (original):

M1=0.972 (fraction of A overlapping B)

M2=0.985 (fraction of B overlapping A)

Manders' Coefficients (using threshold value of 605 for imgA and 1418 for imgB):

M1=0.259 (fraction of A overlapping B)

M2=0.25 (fraction of B overlapping A)

**************************************************

Image A: C1-q4-2-SR__AiryscanProcessed.czi

Image B: C2-q4-2-SR__AiryscanProcessed.czi

Pearson's Coefficient:

r=0.692

Manders' Coefficients (original):

M1=0.981 (fraction of A overlapping B)

M2=0.992 (fraction of B overlapping A)

Manders' Coefficients (using threshold value of 927 for imgA and 1827 for imgB):

M1=0.297 (fraction of A overlapping B)

M2=0.261 (fraction of B overlapping A)

**************************************************

Image A: C1-q4-3-SR__AiryscanProcessed.czi

Image B: C2-q4-3-SR__AiryscanProcessed.czi

Pearson's Coefficient:

r=0.763

Manders' Coefficients (original):

M1=0.976 (fraction of A overlapping B)

M2=0.992 (fraction of B overlapping A)

Manders' Coefficients (using threshold value of 1486 for imgA and 3344 for imgB):

M1=0.306 (fraction of A overlapping B)

M2=0.317 (fraction of B overlapping A)

**************************************************

Image A: C1-q5-1-SR__AiryscanProcessed.czi

Image B: C2-q5-1-SR__AiryscanProcessed.czi

Pearson's Coefficient:

r=0.71

Manders' Coefficients (original):

M1=0.959 (fraction of A overlapping B)

M2=0.987 (fraction of B overlapping A)

Manders' Coefficients (using threshold value of 1294 for imgA and 1878 for imgB):

M1=0.359 (fraction of A overlapping B)

M2=0.098 (fraction of B overlapping A)

**************************************************

Image A: C1-q5-2-SR__AiryscanProcessed.czi

Image B: C2-q5-2-SR__AiryscanProcessed.czi

Pearson's Coefficient:

r=0.699

Manders' Coefficients (original):

M1=0.977 (fraction of A overlapping B)

M2=0.989 (fraction of B overlapping A)

Manders' Coefficients (using threshold value of 1655 for imgA and 1731 for imgB):

M1=0.274 (fraction of A overlapping B)

M2=0.676 (fraction of B overlapping A)

**20250814-S2**

**************************************************

Image A: C1-q2-2-SR__AiryscanProcessed-1.czi

Image B: C2-q2-2-SR__AiryscanProcessed-1.czi

Pearson's Coefficient:

r=0.7

Manders' Coefficients (original):

M1=0.969 (fraction of A overlapping B)

M2=0.995 (fraction of B overlapping A)

Manders' Coefficients (using threshold value of 883 for imgA and 1311 for imgB):

M1=0.334 (fraction of A overlapping B)

M2=0.282 (fraction of B overlapping A)

**************************************************

Image A: C1-q2-2-SR__AiryscanProcessed.czi

Image B: C2-q2-2-SR__AiryscanProcessed.czi

Pearson's Coefficient:

r=0.517

Manders' Coefficients (original):

M1=0.923 (fraction of A overlapping B)

M2=0.988 (fraction of B overlapping A)

Manders' Coefficients (using threshold value of 556 for imgA and 501 for imgB):

M1=0.227 (fraction of A overlapping B)

M2=0.171 (fraction of B overlapping A)
