# Supplementary material for: Expansion Microscopy Reveals the Spatial Association of Therapeutic Antibody–Receptor Complexes With Cholesterol‐Rich Membrane Domains
Source: Angew Chem Int Ed Engl. 2026 Aug 24;65(42):e1206161. doi: 10.1002/anie.1206161 (PMC13653619; doi:10.1002/anie.1206161)
Supplement: Supplementary file 3 [file ANIE-65-e1206161-s003.zip › Raw dara of Manders analysis of CD20 and CD38 and CD45/RTX-D4 raw data in total.docx]

**RTX-CD20 and EGFP-D4**

**20250827-S5**

**************************************************

Image A: C1-q1-2-SR__AiryscanProcessed.czi

Image B: C2-q1-2-SR__AiryscanProcessed.czi

Pearson's Coefficient:

r=0.687

Manders' Coefficients (original):

M1=0.948 (fraction of A overlapping B)

M2=0.952 (fraction of B overlapping A)

Manders' Coefficients (using threshold value of 325 for imgA and 2711 for imgB):

M1=0.434 (fraction of A overlapping B)

M2=0.391 (fraction of B overlapping A)

**************************************************

Image A: C1-q1-3-SR__AiryscanProcessed.czi

Image B: C2-q1-3-SR__AiryscanProcessed.czi

Pearson's Coefficient:

r=0.543

Manders' Coefficients (original):

M1=0.919 (fraction of A overlapping B)

M2=0.949 (fraction of B overlapping A)

Manders' Coefficients (using threshold value of 438 for imgA and 671 for imgB):

M1=0.331 (fraction of A overlapping B)

M2=0.194 (fraction of B overlapping A)

**************************************************

Image A: C1-q4-1-SR__AiryscanProcessed.czi

Image B: C2-q4-1-SR__AiryscanProcessed.czi

Pearson's Coefficient:

r=0.49

Manders' Coefficients (original):

M1=0.878 (fraction of A overlapping B)

M2=0.906 (fraction of B overlapping A)

Manders' Coefficients (using threshold value of 315 for imgA and 350 for imgB):

M1=0.429 (fraction of A overlapping B)

M2=0.187 (fraction of B overlapping A)

**************************************************

Image A: C1-q4-2-SR__AiryscanProcessed.czi

Image B: C2-q4-2-SR__AiryscanProcessed.czi

Pearson's Coefficient:

r=0.551

Manders' Coefficients (original):

M1=0.947 (fraction of A overlapping B)

M2=0.95 (fraction of B overlapping A)

Manders' Coefficients (using threshold value of 480 for imgA and 897 for imgB):

M1=0.374 (fraction of A overlapping B)

M2=0.182 (fraction of B overlapping A)

**************************************************

Image A: C1-q5-1-SR__AiryscanProcessed.czi

Image B: C2-q5-1-SR__AiryscanProcessed.czi

Pearson's Coefficient:

r=0.561

Manders' Coefficients (original):

M1=0.956 (fraction of A overlapping B)

M2=0.967 (fraction of B overlapping A)

Manders' Coefficients (using threshold value of 174 for imgA and 746 for imgB):

M1=0.243 (fraction of A overlapping B)

M2=0.419 (fraction of B overlapping A)

**************************************************

Image A: C1-q5-3-SR__AiryscanProcessed.czi

Image B: C2-q5-3-SR__AiryscanProcessed.czi

Pearson's Coefficient:

r=0.635

Manders' Coefficients (original):

M1=0.926 (fraction of A overlapping B)

M2=0.949 (fraction of B overlapping A)

Manders' Coefficients (using threshold value of 455 for imgA and 1102 for imgB):

M1=0.481 (fraction of A overlapping B)

M2=0.314 (fraction of B overlapping A)

**************************************************

**************************************************

Image A: C1-q7-1-SR__AiryscanProcessed-1.czi

Image B: C2-q7-1-SR__AiryscanProcessed-1.czi

Pearson's Coefficient:

r=0.52

Manders' Coefficients (original):

M1=0.932 (fraction of A overlapping B)

M2=0.941 (fraction of B overlapping A)

Manders' Coefficients (using threshold value of 295 for imgA and 2644 for imgB):

M1=0.304 (fraction of A overlapping B)

M2=0.184 (fraction of B overlapping A)

**************************************************

Image A: C1-q7-2-SR__AiryscanProcessed.czi

Image B: C2-q7-2-SR__AiryscanProcessed.czi

Pearson's Coefficient:

r=0.556

Manders' Coefficients (original):

M1=0.928 (fraction of A overlapping B)

M2=0.942 (fraction of B overlapping A)

Manders' Coefficients (using threshold value of 314 for imgA and 1148 for imgB):

M1=0.251 (fraction of A overlapping B)

M2=0.283 (fraction of B overlapping A)

**************************************************

Image A: C1-q8-1-SR__AiryscanProcessed.czi

Image B: C2-q8-1-SR__AiryscanProcessed.czi

Pearson's Coefficient:

r=0.569

Manders' Coefficients (original):

M1=0.954 (fraction of A overlapping B)

M2=0.95 (fraction of B overlapping A)

Manders' Coefficients (using threshold value of 462 for imgA and 1197 for imgB):

M1=0.468 (fraction of A overlapping B)

M2=0.192 (fraction of B overlapping A)

**************************************************

Image A: C1-q8-2-SR__AiryscanProcessed.czi

Image B: C2-q8-2-SR__AiryscanProcessed.czi

Pearson's Coefficient:

r=0.695

Manders' Coefficients (original):

M1=0.975 (fraction of A overlapping B)

M2=0.973 (fraction of B overlapping A)

Manders' Coefficients (using threshold value of 563 for imgA and 1260 for imgB):

M1=0.561 (fraction of A overlapping B)

M2=0.382 (fraction of B overlapping A)

**************************************************

**************************************************

Image A: C1-q1-1-all 40x water objective.czi

Image B: C2-q1-1-all 40x water objective.czi

Pearson's Coefficient:

r=0.244

Manders' Coefficients (original):

M1=0.518 (fraction of A overlapping B)

M2=0.214 (fraction of B overlapping A)

Manders' Coefficients (using threshold value of 8 for imgA and 25 for imgB):

M1=0.208 (fraction of A overlapping B)

M2=0.159 (fraction of B overlapping A)

**20250827-S6**

**************************************************

Image A: C1-q1-2-SR__AiryscanProcessed-1.czi

Image B: C2-q1-2-SR__AiryscanProcessed-1.czi

Pearson's Coefficient:

r=0.505

Manders' Coefficients (original):

M1=0.919 (fraction of A overlapping B)

M2=0.93 (fraction of B overlapping A)

Manders' Coefficients (using threshold value of 281 for imgA and 671 for imgB):

M1=0.27 (fraction of A overlapping B)

M2=0.16 (fraction of B overlapping A)

**************************************************

Image A: C1-q1-2-SR__AiryscanProcessed-1.czi

Image B: C2-q1-2-SR__AiryscanProcessed-1.czi

Pearson's Coefficient:

r=0.541

Manders' Coefficients (original):

M1=0.972 (fraction of A overlapping B)

M2=0.952 (fraction of B overlapping A)

Manders' Coefficients (using threshold value of 207 for imgA and 858 for imgB):

M1=0.453 (fraction of A overlapping B)

M2=0.304 (fraction of B overlapping A)

**************************************************

Image A: C1-q1-1-40x water objective-SR__AiryscanProcessed.czi

Image B: C2-q1-1-40x water objective-SR__AiryscanProcessed.czi

Pearson's Coefficient:

r=0.617

Manders' Coefficients (original):

M1=0.951 (fraction of A overlapping B)

M2=0.952 (fraction of B overlapping A)

Manders' Coefficients (using threshold value of 486 for imgA and 1315 for imgB):

M1=0.373 (fraction of A overlapping B)

M2=0.361 (fraction of B overlapping A)

**************************************************

Image A: C1-q1-3-SR__AiryscanProcessed.czi

Image B: C2-q1-3-SR__AiryscanProcessed.czi

Pearson's Coefficient:

r=0.562

Manders' Coefficients (original):

M1=0.931 (fraction of A overlapping B)

M2=0.947 (fraction of B overlapping A)

Manders' Coefficients (using threshold value of 190 for imgA and 768 for imgB):

M1=0.287 (fraction of A overlapping B)

M2=0.431 (fraction of B overlapping A)

**************************************************

Image A: C1-q1-4-SR__AiryscanProcessed-1.czi

Image B: C2-q1-4-SR__AiryscanProcessed-1.czi

Pearson's Coefficient:

r=0.622

Manders' Coefficients (original):

M1=0.917 (fraction of A overlapping B)

M2=0.961 (fraction of B overlapping A)

Manders' Coefficients (using threshold value of 195 for imgA and 912 for imgB):

M1=0.433 (fraction of A overlapping B)

M2=0.481 (fraction of B overlapping A)

**************************************************

Image A: C1-q1-4-SR__AiryscanProcessed-1.czi

Image B: C2-q1-4-SR__AiryscanProcessed-1.czi

Pearson's Coefficient:

r=0.468

Manders' Coefficients (original):

M1=0.913 (fraction of A overlapping B)

M2=0.921 (fraction of B overlapping A)

Manders' Coefficients (using threshold value of 196 for imgA and 615 for imgB):

M1=0.37 (fraction of A overlapping B)

M2=0.249 (fraction of B overlapping A)

20250827-S7

**************************************************

Image A: C1-q4-1-SR__AiryscanProcessed-1.czi

Image B: C2-q4-1-SR__AiryscanProcessed-1.czi

Pearson's Coefficient:

r=0.659

Manders' Coefficients (original):

M1=0.963 (fraction of A overlapping B)

M2=0.978 (fraction of B overlapping A)

Manders' Coefficients (using threshold value of 403 for imgA and 1191 for imgB):

M1=0.383 (fraction of A overlapping B)

M2=0.334 (fraction of B overlapping A)

**************************************************

Image A: C1-q4-1-SR__AiryscanProcessed.czi

Image B: C2-q4-1-SR__AiryscanProcessed.czi

Pearson's Coefficient:

r=0.532

Manders' Coefficients (original):

M1=0.954 (fraction of A overlapping B)

M2=0.969 (fraction of B overlapping A)

Manders' Coefficients (using threshold value of 401 for imgA and 1164 for imgB):

M1=0.398 (fraction of A overlapping B)

M2=0.115 (fraction of B overlapping A)

**************************************************

The raw data of three images (M1: 0.294, 0.206, 0.423) were not saved.

**************************************************

Image A: C1-q6-1-SR__AiryscanProcessed.czi

Image B: C2-q6-1-SR__AiryscanProcessed.czi

Pearson's Coefficient:

r=0.592

Manders' Coefficients (original):

M1=0.905 (fraction of A overlapping B)

M2=0.933 (fraction of B overlapping A)

Manders' Coefficients (using threshold value of 303 for imgA and 1272 for imgB):

M1=0.347 (fraction of A overlapping B)

M2=0.276 (fraction of B overlapping A)

20250801-S3

**************************************************

Image A: C1-q3-1-63x oil objective-SR__AiryscanProcessed.czi

Image B: C2-q3-1-63x oil objective-SR__AiryscanProcessed.czi

Pearson's Coefficient:

r=0.538

Manders' Coefficients (original):

M1=0.669 (fraction of A overlapping B)

M2=0.958 (fraction of B overlapping A)

Manders' Coefficients (using threshold value of 278 for imgA and 54 for imgB):

M1=0.468 (fraction of A overlapping B)

M2=0.566 (fraction of B overlapping A)

**************************************************

Image A: C1-q3-6-40x water objective-SR__AiryscanProcessed-1.czi

Image B: C2-q3-6-40x water objective-SR__AiryscanProcessed-1.czi

Pearson's Coefficient:

r=0.649

Manders' Coefficients (original):

M1=0.91 (fraction of A overlapping B)

M2=0.982 (fraction of B overlapping A)

Manders' Coefficients (using threshold value of 1265 for imgA and 851 for imgB):

M1=0.398 (fraction of A overlapping B)

M2=0.231 (fraction of B overlapping A)

**************************************************

Image A: C1-q3-6-40x water objective-SR__AiryscanProcessed-1.czi

Image B: C2-q3-6-40x water objective-SR__AiryscanProcessed-1.czi

Pearson's Coefficient:

r=0.62

Manders' Coefficients (original):

M1=0.897 (fraction of A overlapping B)

M2=0.962 (fraction of B overlapping A)

Manders' Coefficients (using threshold value of 612 for imgA and 1445 for imgB):

M1=0.398 (fraction of A overlapping B)

M2=0.362 (fraction of B overlapping A)

**************************************************

Image A: C1-q3-6-40x water objective-SR__AiryscanProcessed-1.czi

Image B: C2-q3-6-40x water objective-SR__AiryscanProcessed-1.czi

Pearson's Coefficient:

r=0.62

Manders' Coefficients (original):

M1=0.897 (fraction of A overlapping B)

M2=0.962 (fraction of B overlapping A)

Manders' Coefficients (using threshold value of 612 for imgA and 1141 for imgB):

M1=0.517 (fraction of A overlapping B)

M2=0.327 (fraction of B overlapping A)

**************************************************

Image A: C1-q3-7-40x water objective-SR__AiryscanProcessed-1.czi

Image B: C2-q3-7-40x water objective-SR__AiryscanProcessed-1.czi

Pearson's Coefficient:

r=0.661

Manders' Coefficients (original):

M1=0.947 (fraction of A overlapping B)

M2=0.982 (fraction of B overlapping A)

Manders' Coefficients (using threshold value of 940 for imgA and 1550 for imgB):

M1=0.269 (fraction of A overlapping B)

M2=0.525 (fraction of B overlapping A)

**************************************************

Image A: C1-q3-7-40x water objective-SR__AiryscanProcessed-1.czi

Image B: C2-q3-7-40x water objective-SR__AiryscanProcessed-1.czi

Pearson's Coefficient:

r=0.661

Manders' Coefficients (original):

M1=0.947 (fraction of A overlapping B)

M2=0.982 (fraction of B overlapping A)

Manders' Coefficients (using threshold value of 940 for imgA and 1224 for imgB):

M1=0.365 (fraction of A overlapping B)

M2=0.476 (fraction of B overlapping A)

**************************************************

Image A: C1-q3-7-40x water objective-SR__AiryscanProcessed-1.czi

Image B: C2-q3-7-40x water objective-SR__AiryscanProcessed-1.czi

Pearson's Coefficient:

r=0.616

Manders' Coefficients (original):

M1=0.931 (fraction of A overlapping B)

M2=0.976 (fraction of B overlapping A)

Manders' Coefficients (using threshold value of 742 for imgA and 709 for imgB):

M1=0.506 (fraction of A overlapping B)

M2=0.293 (fraction of B overlapping A)

**************************************************

Image A: C1-q4-1-40x water objective-SR__AiryscanProcessed-1.czi

Image B: C2-q4-1-40x water objective-SR__AiryscanProcessed-1.czi

Pearson's Coefficient:

r=0.661

Manders' Coefficients (original):

M1=0.916 (fraction of A overlapping B)

M2=0.981 (fraction of B overlapping A)

Manders' Coefficients (using threshold value of 1185 for imgA and 2399 for imgB):

M1=0.257 (fraction of A overlapping B)

M2=0.285 (fraction of B overlapping A)

**************************************************

Image A: C1-q4-1-40x water objective-SR__AiryscanProcessed-1.czi

Image B: C2-q4-1-40x water objective-SR__AiryscanProcessed-1.czi

Pearson's Coefficient:

r=0.499

Manders' Coefficients (original):

M1=0.855 (fraction of A overlapping B)

M2=0.963 (fraction of B overlapping A)

Manders' Coefficients (using threshold value of 643 for imgA and 687 for imgB):

M1=0.332 (fraction of A overlapping B)

M2=0.269 (fraction of B overlapping A)

**************************************************

Image A: C1-q4-2-40x water objective-SR__AiryscanProcessed.czi

Image B: C2-q4-2-40x water objective-SR__AiryscanProcessed.czi

Pearson's Coefficient:

r=0.606

Manders' Coefficients (original):

M1=0.917 (fraction of A overlapping B)

M2=0.985 (fraction of B overlapping A)

Manders' Coefficients (using threshold value of 786 for imgA and 1045 for imgB):

M1=0.333 (fraction of A overlapping B)

M2=0.406 (fraction of B overlapping A)

**************************************************

Image A: C1-q4-3-40x water objective-SR__AiryscanProcessed-1.czi

Image B: C2-q4-3-40x water objective-SR__AiryscanProcessed-1.czi

Pearson's Coefficient:

r=0.636

Manders' Coefficients (original):

M1=0.914 (fraction of A overlapping B)

M2=0.986 (fraction of B overlapping A)

Manders' Coefficients (using threshold value of 1414 for imgA and 632 for imgB):

M1=0.43 (fraction of A overlapping B)

M2=0.192 (fraction of B overlapping A)

**************************************************

Image A: C1-q4-3-40x water objective-SR__AiryscanProcessed.czi

Image B: C2-q4-3-40x water objective-SR__AiryscanProcessed.czi

Pearson's Coefficient:

r=0.59

Manders' Coefficients (original):

M1=0.882 (fraction of A overlapping B)

M2=0.98 (fraction of B overlapping A)

Manders' Coefficients (using threshold value of 703 for imgA and 954 for imgB):

M1=0.297 (fraction of A overlapping B)

M2=0.238 (fraction of B overlapping A)
